# Rational Synthesis and Regulation of Hollow Structural Materials for Electrocatalytic Nitrogen Reduction Reaction

**DOI:** 10.1002/advs.202104183

**Published:** 2021-12-10

**Authors:** Cong Xue, Xinru Zhou, Xiaohan Li, Nan Yang, Xue Xin, Yusheng Wang, Weina Zhang, Jiansheng Wu, Wenjing Liu, Fengwei Huo

**Affiliations:** ^1^ Key Laboratory of Flexible Electronics (KLOFE) and Institute of Advanced Materials (IAM) Nanjing Tech University 30 South Puzhu Road Nanjing 211816 China

**Keywords:** electrocatalysis, hollow structural materials, nitrogen reduction reaction, regulation strategies, synthetic methods

## Abstract

The electrocatalytic nitrogen reduction reaction (NRR) is known as a promising mean of nitrogen fixation to mitigate the energy crisis and facilitate fertilizer production under mild circumstances. For electrocatalytic reactions, the design of efficient catalysts is conducive to reducing activation energy and accelerating lethargic dynamics. Among them, hollow structural materials possess cavities in their structures, which can slack off the escape rate of N_2_ and reaction intermediates, prolong the residence time of N_2_, enrich the reaction intermediates’ concentration, and shorten electron transportation path, thereby further enhancing their NRR activity. Here, the basic synthetic strategies of hollow structural materials are introduced first. Then, the recent breakthroughs in hollow structural materials as NRR catalysts are reviewed from the perspective of intrinsic, mesoscopic, and microscopic regulations, aiming to discuss how structures affect and improve the catalytic performance. Finally, the future research directions of hollow structural materials as NRR catalysts are discussed. This review is expected to provide an outlook for optimizing hollow structural NRR catalysts.

## Graphical abstract

Recent progresses about the advantages of hollow structural materials for nitrogen reduction reaction are summarized. Besides, the top‐down fine regulating strategies from the perspective of intrinsic, mesoscopic, and microscopic regulations are introduced, aiming to discuss how various structures affect and improve the catalytic performance. These outputs render hollow structural materials to be the centerpiece for various applications, especially energy conversion applications.

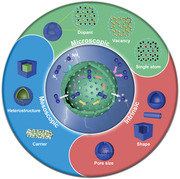

## 1 Introduction

Ammonia (NH_3_) is not only widely used in the manufacture of fertilizers, pharmaceuticals, and chemical industries,^[^ ^1^, ^2^ ^]^ but also is considered as a promising energy carrier because of its high hydrogen content (17.8 wt%), easy liquefaction, and convenient transportation.^[^ ^3^, ^4^ ^]^ For over a century, NH_3_ production has relied heavily on the traditional Haber–Bosch method. This method is considered to be one of the most significant invention of the 20th century as it triggers the revolution of modern agriculture and provided food security for population growth (at least half of the nitrogen in the human body is currently derived from synthetic NH_3_). However, the hydrogen as reagent in this method is mainly produced by steam reforming using natural gas or fossil fuels, and the reaction process is carried out at high temperature and high pressure, which consumes plenty of energy (about 1–2% of the total global energy consumption) and releases a large amount of CO_2_ (about 1% of the global annual CO_2_ emissions).^[^ ^5^, ^6^ ^]^ Considering energy shortages and increasing environmental problems, it is urgent to develop an energy‐saving, environmentally friendly, green, and sustainable method to replace the traditional Haber–Bosch method to fix nitrogen.

Various strategies on N_2_ fixation under mild conditions have been developed in recent years, such as mimicking biological nitrogenase,^[^ ^7^, ^8^ ^]^ photocatalytic nitrogen fixation,^[^ ^9^, ^10^ ^]^ and electrocatalytic nitrogen fixation.^[^ ^11^, ^12^ ^]^ Compared with the traditional resource‐intensive Haber–Bosch method, electrocatalytic nitrogen reduction reaction (NRR) shows great potential to realize small‐scale, distributed, and even on‐site direct synthesis of NH_3_ by utilizing water as a hydrogen source and renewable energy such as wind, solar, and tidal energy as power sources. In this way, the synthesized NH_3_ can be further converted to supply crops, thereby saving the cost of nitrogen during transportation and storage.^[^ ^13^, ^14^, ^15^ ^]^ In practice, the limitation of N_2_ activation prompts us to find a suitable and efficient way to break its inert bonds. Concurrently, an inevitable issue is the competitive hydrogen evolution reaction (HER) in the aqueous electrolyte, which will cause the reducing the Faradaic efficiency (FE).^[^ ^14^, ^16^ ^]^

In order to improve catalytic activity and selectivity, the development of efficient catalysts is essential for electrocatalytic nitrogen fixation. Currently, researchers have made enormous efforts in designing catalysts, including noble metal catalysts, non‐noble metal catalysts, and nonmetal catalysts.^[^ ^17^, ^18^, ^19^ ^]^ In addition, many effective strategies have been developed to regulate the physical and chemical properties of catalysts to enhance their catalytic activity. For example, the structure and morphology of these materials (2D flakes, porous structure, hollow structure, etc.) were optimized to increase the apparent activity,^[^ ^20^, ^21^ ^]^ defects (doping, vacancy, amorphous, etc.) were created to improve the intrinsic activity,^[^ ^22^, ^23^ ^]^ various synthetic strategies (in situ synthesis method, immersion reduction method, electrodeposition method, etc.) were adopted to obtain the catalysts grown on the electrode to reduce interface resistance,^[^ ^24^, ^25^ ^]^ compounds were loaded with some other conductive materials (carbon black, graphene, carbon nanotubes, etc.) to promote charge transfer,^[^ ^26^, ^27^ ^]^ etc.

Among them, hollow structural materials, a category of structures consisting of defined shell layers and internal cavity, have received extensive attention due to their unique structure and excellent performance according to the following advantages. First, considering the issue of weak solubility of N_2_ in aqueous electrolytes, the hollow structural materials can give full play to their structural merits. The hollow structural cavity can be used as a nanoreactor to restrict the reactants, prolong the residence time of N_2_, increase the collision probability of N_2_, and improve the contact between N_2_ and active sites, thereby expediting the reaction rate.^[^ ^28^ ^]^ Meanwhile, this restriction increases the concentration of local reaction intermediates in the cavities to accelerate further reactions and improve the NRR reaction kinetics.^[^ ^29^, ^30^ ^]^ Second, sufficient exposure of active sites is key to efficiently catalyzing the conversion of N_2_ to NH_3_. No matter it is the capping agents used in the synthesis of the catalysts, or the polymer binders (Nafion) required to support the catalysts on the reactive electrode, the exposure of active sites is inevitably affected.^[^ ^31^ ^]^ However, the cavities in the hollow structural materials are less affected by these factors and can better expose the active sites. Third, the N_2_ diffusion velocity is also a key step of NRR. The tightly connected cavities of the hollow structural materials and the gaps between different structures have high permeability, which can provide a fast diffusion channel for N_2_.^[^ ^32^ ^]^ Last but not the least, catalytic durability is an important indicator for evaluating the performance of catalysts. The self‐supporting 3D structure of the hollow material has excellent structural stability and helps to obtain good catalytic durability.^[^ ^33^, ^34^ ^]^ In addition, various strategies have been developed to optimize the apparent activity and intrinsic activity of hollow structural materials to further improve their catalytic performance. In short, hollow structural materials have attracted much attention as excellent catalyst candidates and have been widely used in electrocatalytic reactions.

**Scheme 1:**
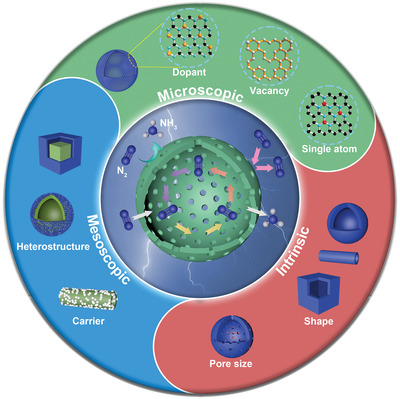
Schematic diagram of hollow structural catalysts for NRR. Hollow structural materials according to the regulation strategy toward NRR are divided into three main sections: 1) intrinsic regulation (shape, pore size); 2) mesoscopic regulation (loading nanomaterials, construction of heterostructures); 3) microscopic regulation (doping engineering, vacancy engineering, single atom engineering, etc.).

This review focuses on the latest development of hollow structural materials for nitrogen reduction reaction under environmental conditions. First, the reaction mechanisms of NRR will be introduced. Second, the common synthetic methods of hollow structural materials are summarized and divided into three categories: hard template method, soft template method, and self‐template/template‐free method. Then, a comprehensive introduction of NRR catalysts according to the sizes and types of catalysts is summarized from the following three aspects: 1) intrinsic regulation of hollow structural catalysts, focusing on the influence of shape and pore size of the hollow structural materials on the catalytic performance; 2) mesoscopic regulation of hollow structural catalysts, focusing on the combination of hollow structural materials and other molecular ingredients on NRR catalytic performance; 3) microscopic regulation of hollow structural catalysts, focusing on the influence of dopant, vacancy, and single‐atom regulation on the active site modification of the hollow structural catalysts (**Scheme** **1**). Finally, challenges and possible solutions faced by hollow structural materials as NRR electrocatalysts will be proposed. It is anticipated that this review will provide valuable insights into the synthesis and design of the next‐generation efficient hollow structural NRR catalysts.

## 2 Reaction Mechanisms of NRR

**Figure 1:**
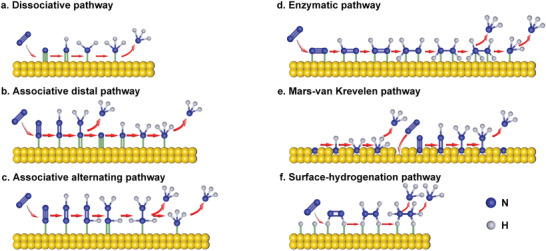
a–f) Schematic diagram of possible reaction mechanisms for NRR on the catalyst surface. The blue balls represent nitrogen atoms, and the gray balls represent hydrogen atoms.

In general, NRR occurs in three steps: i) adsorption of N_2_ on active sites, ii) hydrogenation of the adsorbed N_2_, iii) NH_3_ desorption. Specifically, according to the different sequence of N≡N cleavage and hydrogenation, two possible mechanisms have been proposed: i) dissociative pathway, ii) associative pathway.^[^ ^35^ ^]^ As shown in **Figure** **1**a, in the dissociative pathway, N≡N cleavage occurs before hydrogenation. The N≡N triple bond in nitrogen is dissociated first, and then the cracked N adsorbs on the catalyst surface and hydrogenates inch by inch until NH_3_ is generated and released. Since extremely high energy is required for breaking N≡N, the dissociative pathway is mainly found in the Haber–Bosch process of industrial nitrogen fixation with high energy consumption. For associative pathway, N≡N cleavage occurs in the hydrogenation process and can be divided into i) associative distal pathway and ii) associative alternating pathway according to the different hydrogenation sequences of two N atoms. As shown in Figure 1b, in the associative distal pathway, one end of the N_2_ molecule is adsorbed on the catalyst surface, and the N atom far away from the catalyst surface is hydrogenated first until NH_3_ is generated and released. Subsequently, the remaining N atom undergoes the same procedure and is released. Differently, in the associative alternating pathway (Figure 1c), the hydrogen atoms alternately combine with two N atoms until NH_3_ is released, despite that one end of the N_2_ molecule is adsorbed on the surface of the catalyst just like that in the associative distal pathway. In addition to the abovementioned cases where only one N atom in the N_2_ molecule is adsorbed on the surface of the catalyst, an enzymatic pathway has been proposed in which two N atoms of the N_2_ molecule are adsorbed on the surface of the catalyst simultaneously.^[^ ^36^ ^]^ As shown in Figure 1d, in the enzymatic pathway, the two N atoms adsorbed on the surface of the catalyst are hydrogenated sequentially, and finally NH_3_ is generated and released.

Furthermore, a new mechanism Mars–van Krevelen (MvK) pathway that occurs on transition metal nitrides has been proposed by Abghoui and Skúlason and verified by theoretical calculations.^[^ ^37^, ^38^, ^39^, ^40^ ^]^ The adsorption of N_2_ molecules on the catalyst surface needs to undergo endothermic process and the N_2_ dissociation also needs to overcome a very strong barrier, making the dissociative pathway difficult to occur. The hydrogenation of *N_2_ to *N_2_H requires a large bias voltage, which is not favorable for the associative pathway. Although the associative–dissociative mixed pathway may also occur, MvK on transition metal nitrides occurs most favorably because the MvK pathway on metal nitrides has the lowest overpotential. In the MvK pathway, unlike the conventional one, hydrogenation first takes place on the lattice nitrogen on the surface of the transition metal nitrides until NH_3_ is formed and released. Subsequently, the resulting nitrogen vacancies chemically adsorb N_2_ molecules and follow the associative distal pathway for hydrogenation (Figure 1e).

Although many studies have shown that N_2_ molecules can achieve the electrochemical conversion from N_2_ to NH_3_ at very low potential on noble metals. However, none of the existing NRR reaction pathways are suitable for these catalysts, whether dissociative pathway or associative pathway. Wang and co‐workers discovered a novel NRR mechanism called surface‐hydrogenation mechanism through first principle calculations.^[^ ^41^ ^]^ Unlike the traditional reaction pathway, H^+^ reduction occurs first on the noble metal catalyst surface instead of N_2_ adsorption. It is proposed to explain the differences between experimental and computational methods. First, H^+^ is reduced to *H, which is the potential determining step (PDS). Second, N_2_ is activated and reduced to *H_2_N_2_, which is considered as a rate‐determining step. Subsequently, *H_2_N_2_ is further reduced to NH_3_ and released (Figure 1f). Moreover, proton supply is particularly critical for the catalytic activity of NRR during proton‐coupled electron transfer (PCET) reaction. Some studies have shown that pH values can affect proton supply, leading to selectivity differences.^[^ ^42^, ^43^ ^]^ In addition to pH values, Yin and co‐workers found that some alkali metal cations (Li^+^, Na^+^, K^+^, and Cs^+^) can regulate the proton transfer rate to inhibit HER and increase catalyst selectivity.^[^ ^44^ ^]^ It can be seen that although theoretical calculation has become a powerful means to judge the reaction mechanism, it is generally based on idealized models, inevitably ignoring many factors, so it is essential to combine theory with experiment. At the same time, there is an urgent need to develop and apply in situ test and characterization methods to accurately define the reaction process.

## 3 Synthetic Methods for Hollow Structural Materials

In the electrocatalysis field, the unique architecture of hollow materials endows them with potential catalytic possibilities. For example, the well‐defined cavity could slack off the escape rate of N_2_ and reaction intermediates, the high surface area could provide abundant exposure of active sites, the low density reduces the mass and charge transfer diffusion length. Therefore, the catalytic activity of hollow materials is generally affected by their structure and composition. In order to obtain better catalytic performance, it is necessary to carefully design hollow structural materials. Hence, it is critical to understand and choose the appropriate method for controllable synthesis. This section will summarize the synthetic methods of hollow structural materials from three categories: hard template method, soft template method, and self‐template/template‐free method to better understand the synthetic methods of hollow structural materials and characteristics of variform approaches.

### 3.1 Hard Template Method

The hard template method, as the name suggests, uses rigid material as the template to be covered with the prepared material in the outer layer. Then, the desired hollow structural materials are obtained by removing the template. Generally speaking, the shapes of the hollow structural materials ultimately depend on the shape of the template. The parameters of hollow structural materials, such as the size, shell thickness, shell number, and pore size, can be controlled by adjusting the raw material concentration, pH values, reaction temperature, reaction time, and other conditions. It is worth noting that materials with poor chemical stability and high temperature instability are difficult to be developed by hard template method because the removal of the template inevitably requires the use of etching or high temperature calcination. After more than 20 years of development, the kinds of common hard template materials are enriched, including polystyrene (PS) balls, silica, carbon balls, metal particles, etc.

**Figure 2:**
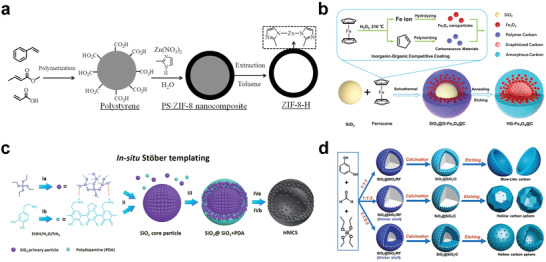
Synthesis of hollow structural materials via hard template method. a) Schematic diagram of fabrication of ZIF‐8‐H. Reproduced with permission.^[^ ^45^ ^]^ Copyright 2014, American Chemical Society. b) Schematic illustration for the synthesis of the hollow gradient structured Fe_3_O_4_@C nanospheres by an inorganic–organic competitive coating strategy. Reproduced with permission.^[^ ^46^ ^]^ Copyright 2021, Springer Nature. c) Schematic diagram of the in situ Stöber templating of HMCS. Reproduced with permission.^[^ ^52^ ^]^ Copyright 2016, Royal Society of Chemistry. d) Scheme of the synthetic process of HBC or hollow carbon sphere (HCS) with different shell thicknesses via adjusting mass ratio of reagents. Reproduced with permission.^[^ ^53^ ^]^ Copyright 2020, Wiley‐VCH.

Li and co‐workers developed a hollow zeolitic imidazolate framework‐8 nanosphere (ZIF‐8‐H) based on the hard template method.^[^ ^45^ ^]^ First, carboxylate‐terminated PS nanospheres were synthesized as hard templates, and then ZIF‐8 was grown on the surface of the spheres through the coordination of COOH functional groups with Zn ions and 2‐methylimidazole. Finally, toluene was used to remove the PS template to obtain ZIF‐8‐H (**Figure** **2**a). Recently, Zhao and co‐workers prepared Fe_3_O_4_–C nanospheres with a hollow gradient structure (HG‐Fe_3_O_4_@C) through an organic–inorganic competitive coating strategy by using SiO_2_ as the hard template.^[^ ^46^ ^]^ In their strategy, ferrocene could be hydrolyzed in solvent to form Fe_3_O_4_, and it could also be polymerized to form carbon. The formed Fe_3_O_4_ and carbon would compete with each other and be deposited on the surface of SiO_2_ to obtain SiO_2_@G‐Fe_3_O_4_@C. Then, the as‐synthesized materials were treated by high temperature and etching to remove SiO_2_ to obtain HG‐Fe_3_O_4_@C (Figure 2b). In addition to hollow nanosphere, for example, nanofibers,^[^ ^47^, ^48^ ^]^ nanotube arrays,^[^ ^49^, ^50^ ^]^ and nanocubes,^[^ ^51^ ^]^ were successfully achieved by utilizing SiO_2_, metal oxides, anodic aluminum oxide, and zeolite as the hard templates.

However, it is necessary to synthesize the hard template first, and then coat the required materials on the surface of the template in the abovementioned methods. Considering the cost and the simplification of experimental procedures, some in situ synthetic methods which integrate the template creation with the coating materials as one step have been developed to obtain materials in a convenient way. Yu and co‐workers developed an in situ Stöber template method to prepare hollow mesoporous carbon spheres (HMCSs).^[^ ^52^ ^]^ First, tetraethyl orthosilicate (TEOS) was hydrolyzed and condensed into 2–3 nm SiO_2_ nanoparticles, and then monodispersed SiO_2_ clusters were formed by secondary nucleation (Figure 2c). Subsequently, polydopamine (PDA) oligomers were polymerized by coating dopamine and SiO_2_ nanoparticles were cocondensed to SiO_2_ clusters to form a SiO_2_@SiO_2_/PDA core–shell structure, and then HMCS was obtained by carbonization and NaOH etching. This in situ synthetic method avoids the introduction of surfactants and is therefore more economical. Meanwhile, the pore size and shell thickness of HMCS can be easily adjusted by changing the ratio of reagents and the growth time of SiO_2_ and PDA. Very recently, Fan and co‐workers developed hollow bowl carbon (HBC) structures using similar in situ SiO_2_ template methods (Figure 2d).^[^ ^53^ ^]^ They found that alteration in the ratio of resorcinol (R), formaldehyde (F), and TEOS would determine the final morphology of the materials, in the case where the ratio of the above three compounds was 1:1:1, HBC was successfully formed due to effect of capillary after removing SiO_2_ by hydrofluoric acid etching.

**Figure 3:**
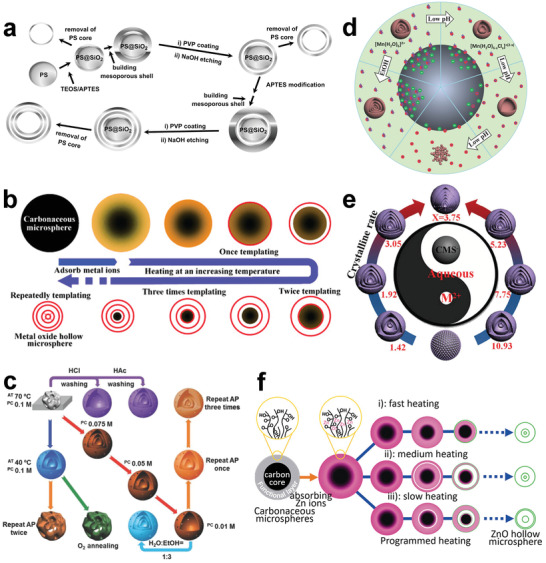
Synthesis of multishelled hollow structural materials via hard template method. a) Schematic diagram of the fabrication of hollow multishelled silica nanospheres. Reproduced with permission.^[^ ^54^ ^]^ Copyright 2011, Elsevier. b) Schematic diagram of the sequential template approach for the synthesis of multiple‐shelled hollow metal oxide microsphere. Reproduced with permission.^[^ ^56^ ^]^ Copyright 2011, Wiley‐VCH. c) Effects of synthetic conditions on the morphology of products. Reproduced with permission.^[^ ^57^ ^]^ Copyright 2016, Springer Nature. d) Scheme of synthetic mechanism for multishelled hollow microspheres and Mn_2_O_3_ nanoparticles under different adsorption conditions. Reproduced under the terms of the Creative Commons CC‐BY license.^[^ ^58^ ^]^ Copyright 2014, The Authors. Published by Wiley‐VCH. e) Control of the shell number of hollow microspheres by tuning the molar ratio of reagents. Reproduced with permission.^[^ ^59^ ^]^ Copyright 2017, Wiley‐VCH. f) Schematic diagram of the generation of multishelled ZnO hollow microspheres via different heating processes. Reproduced with permission.^[^ ^60^ ^]^ Copyright 2012, Wiley‐VCH.

In addition to simple single‐shell hollow structural materials, many complex hollow materials with multishelled structures can also be prepared by the hard template method. For example, Yeh and co‐workers synthesized double‐shelled and triple‐shelled mesoporous SiO_2_ nanospheres via the shell‐by‐shell method.^[^ ^54^ ^]^ They coated SiO_2_ layer by layer on the PS sphere template, and further employed protective etching to prepare nanospheres with different shell layers (**Figure** **3**a). The study found that the introduction of 3‐aminopropyltriethoxysilane is extremely important for the protection of the second layer of SiO_2_, and the introduction of polyvinylpyrrolidone (PVP) playing passivation effect on the second and third layers of SiO_2_ is also key to form multishelled compounds. Although the shell‐by‐shell method is relatively simple in design, and multiple‐shelled layers can also be prepared by repeated steps, the cumbersome steps in practical operation hinder its large‐scale promotion and application. Furthermore, choosing a suitable template to ensure uniform coating of the material also affects the general applicability of the method. To solve these problems, Wang and co‐workers developed a one‐step synthetic method using carbon microspheres (CMSs) as hard templates to prepare multishelled hollow metal oxide microspheres. They prepared a series of spinel ferrites (MFe_2_O_4_, M = Zn, Co, Ni, Cd) using CMSs as sacrificial templates for the first time.^[^ ^55^ ^]^ In 2011, this method was officially named as the sequential template method (Figure 3b).^[^ ^56^ ^]^ Specifically, metals ions absorbed on the surface of CMS template were first accumulated and oxidized until the formation of metal oxide shell by pyrolysis process. As the combustion progressed, the CMS was separated from the metal oxide shell due to the volume of the CMS template being decreased. Subsequently, the remaining CMS could further act as a template and the above process was repeated to form inner shell layers. Different from the traditional hard template method, CMS was used as a carrier for adsorbing metal ions and a continuous template to construct a multishell hollow structure in the sequential template method. In short, this simple and convenient method is very conducive to large‐scale production.

Furthermore, various parameters of multishelled hollow structural materials, such as the shell layer numbers, shell thicknesses, shell spacing, and shell sizes, can be better controlled by adjusting various conditions during the adsorption and heat treatment processes. In the process of adsorbing metal ions, adsorption time, metal ion concentration, adsorption temperature, solvent type, and pH values are all adjustable variables. During heat treatment, the selection of atmosphere, heating rate, and duration time are particularly important. For example, Wang and co‐workers systematically studied the effects of adsorption temperature and time, precursor concentration, solvent composition, heat treatment atmosphere, and other conditions on materials (Figure 3c).^[^ ^57^ ^]^ Another example is that hollow Mn_2_O_3_ microspheres with different numbers of shell layers were prepared, as the pH values increased, the shell layer numbers increased (Figure 3d).^[^ ^58^ ^]^ This is due to the increase in the negative charge on the surface of CMSs as the pH values increases, so that more positively charged Mn ions can be attracted through electrostatic attraction. Finally, under the same conditions, expect the changing pH values; single‐shelled, double‐shelled, and triple‐shelled hollow Mn_2_O_3_ microspheres were obtained at pH values of 3.35, 4.43, and 6.43, respectively. Besides, the shell layer numbers can also be adjusted by controlling the rate of crystallization. For example, the crystallization rate can be adjusted by changing the molar ratios of the reagents to obtain hollow microspheres with different shell layers (Figure 3e).^[^ ^59^ ^]^ Furthermore, the heating rate will affect the speed of template removal and shell spacing. For instance, triple‐shelled ZnO microspheres with different shell spacings were prepared by changing the heating rates (Figure 3f).^[^ ^60^ ^]^ Apart from CMSs, metal–organic frameworks (MOFs),^[^ ^61^, ^62^ ^]^ polymers,^[^ ^63^, ^64^ ^]^ metal carbonates^[^ ^65^ ^]^ can also be used as templates to synthesize hollow multishelled materials by the sequential template method.

### 3.2 Soft Template Method

In addition to materials used in hard template method, micelles/vesicles, emulsions, bubbles, etc., can also act as templates to prepare hollow nanostructures. This method is called soft template method, and is often related with less rigid materials. In contrast to the hard template method that demands hard conditions to remove the template, the soft counterpart only involves simple cleaning or extraction. Under certain circumstances, it is not even necessary to consider the removal of the template. Therefore, the preparation of hollow structural materials by the soft template method is a viable option for those chemically and thermally unstable materials. However, compared with the hard template method, the soft template method shows poorer control over the shape, shell thickness, and size of the hollow structural materials, which is urgently needed to be solved. So far, according to classification of soft templates, the soft template method can be divided into the following categories: 1) micelle/vesicle method, 2) emulsion method, 3) bubble method, and 4) spray method.

**Figure 4:**
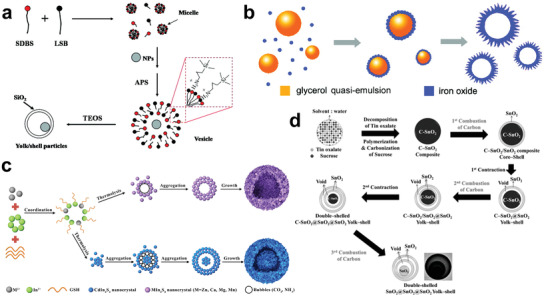
Synthesis of hollow structural materials via soft template method. a) Schematic diagram of the formation yolk/SiO_2_ shell containing movable nanoparticles cores. Reproduced with permission.^[^ ^69^ ^]^ Copyright 2009, American Chemical Society. b) Mechanism of the generation of *α*‐Fe_2_O_3_ hierarchical hollow spheres. Reproduced with permission.^[^ ^71^ ^]^ Copyright 2011, American Chemical Society. c) Schematic illustration of the ternary sulfide hollow and yolk–shell structure formation. Reproduced with permission.^[^ ^80^ ^]^ Copyright 2018, American Chemical Society. d) Scheme of the formation of the SnO_2_ yolk–shell structure with different shell layers. Reproduced with permission.^[^ ^83^ ^]^ Copyright 2013, Wiley‐VCH.

Micelles/vesicles are formed by self‐assembly of amphiphilic molecules with both hydrophilicity and lipophilicity in a single‐phase solvent.^[^ ^66^, ^67^ ^]^ Common amphiphilic molecules are block copolymers and surfactants. The micelles usually have a single‐layer structure with the hydrophilic side facing outward, and the vesicles usually have a double‐layered structure or a multilayer structure with only the hydrophilic side facing outward. The morphology and structure of the micelle/vesicle template can be changed by adjusting the pH values, temperature, concentration of amphiphilic molecules, and the ionic strength of the solution.^[^ ^68^ ^]^ After depositing the targeted material on the hydrophilic or hydrophobic interface of the micelle/vesicle, the hollow structural materials are formed by removing the template. For example, Wu and Xu reported the strategy of synthesizing yolk–shell structure by soft template method first, and then used SiO_2_ nanoparticles to encapsulate the yolk/shell particles (**Figure** **4**a).^[^ ^69^ ^]^ In this work, lauryl sulfonate betaine and sodium dodecyl benzenesulfonate as mixed surfactants and templates were added to the solution forming micelle as nuclear precursor first. After that, 3‐amino‐propyltriethoxysilane (APS) as initiator was added to induce vesicle formation. It is worth noting that the partially protonated APS playing the role of directing agent was electrostatically adsorbed on the vesicles’ surface. Then, TEOS was added to the vesicle and interacted with the terminal functional groups of APS by means of hydrolysis and condensation to form yolk/shell particles with SiO_2_‐encapsulated vesicles. In addition, this excellent template method can also be extended to encapsulate other nanoparticles, such as Au nanoparticles and spindle‐shaped Fe_2_O_3_ particles.

Emulsion method usually refers to two or more immiscible liquids forming an emulsion with the aid of amphiphilic molecules. It has the characteristics of good particle dispersion and thermodynamic stability. The emulsion can be divided into water‐based emulsion/oil‐in‐water and oil‐based emulsion/water‐in‐oil according to the dispersion phase.^[^ ^70^, ^71^, ^72^, ^73^ ^]^ The hollow structural materials can be prepared by depositing the targeted materials on the surface of the emulsion, and then removing the emulsion template by simple washing or calcination. Lou and co‐workers reported the synthesis of *α*‐Fe_2_O_3_ hollow nanospheres using quasiemulsion as a soft template (Figure 4b).^[^ ^71^ ^]^ In their strategy, glycerol was mixed with water to form a quasiemulsion, which was partially polymerized to form an emulsified sphere under hydrothermal conditions, and then the adjunction of Fe precursor was hydrolyzed and aggregated on the surface of the emulsion template. The glycerin quasiemulsion can be simply removed by solvent extraction in the subsequent cleaning process.

The template removal requires reasonable design because improper strategies may not only make it difficult to completely remove the template, but also may bring out collapsing in the structure or the hole. In comparison, the bubble template method is a cost‐effective and convenient method since there is no need to consider template removal. Currently, bubble template formation methods have been developed, including gas blowing method,^[^ ^74^, ^75^ ^]^ chemical reaction method,^[^ ^76^, ^77^ ^]^ and ultrasonic induction method.^[^ ^78^, ^79^ ^]^ For example, Hu and co‐workers synthesized a series of ternary sulfide hollow structural materials using the chemical reaction bubble template method (Figure 4c).^[^ ^80^ ^]^ Glutathione (GSH) as the source of bubble template could coordinate with metal ions M (such as Cd^2+^, Zn^2+^, Ca^2+^, Mg^2+^, and Mn^2+^) and In^3+^ through strong coordination to form complexes. MIn_2_S_4_ crystals were obtained through a further pyrolysis process, and driven by surface energy, the as‐synthesized MIn_2_S_4_ crystals grew on the CO_2_ and NH_3_ bubbles which were released by pyrolysis, and finally ternary sulfide hollow structural materials were synthesized. It is worth noting that the coordination ability of metal ions and GSH is related to the ion radius of metal ions. When the metal ions are Cd^2+^ and In^2+^ with close ionic radius, their coordination ability with GSH will be weak, resulting in the formation of small‐sized CdIn_2_S_4_ single crystals. The assembly speed of the sphere CdIn_2_S_4_ was the fastest because the smaller the single crystal size, the higher the surface energy, and the time of bubble formation would lag behind the assembly of these small‐sized single crystals, leading to the production of yolk–shell structure.

The spray method is considered to be a soft template method with industrial application prospects, because it can continuously produce hollow structural materials on a large scale.^[^ ^81^ ^]^ The spray method can also be regarded as a special sequential template method, except that it does not require the synthesis of carbon microspheres in the first place. The general process of spray method is as follows: the mixed solution of metal salt and sucrose is atomized into droplets by the ultrasonic sprayer, and then the sucrose is polymerized and carbonized through a combustion process to compete with the crystallization of metal to form hollow structural materials.^[^ ^82^ ^]^ For instance, the SnO_2_ yolk–shell structure was synthesized in a simple one‐pot spray method by Kang and co‐workers (Figure 4d).^[^ ^83^ ^]^ In their strategy, tin oxalate and sucrose were dissolved in water as precursors, the atomized droplets were formed under ultrasonic force, and the final carbon–SnO_2_ particles were obtained through further pyrolysis and polymerization. They found that the pyrolyzation temperature of carbon and the concentration of oxygen would affect the morphology of the final product. At low temperature, the slow combustion of carbon and sufficient oxygen supply eventually facilitated the formation a single‐shell SnO_2_@SnO_2_ with yolk–shell structure. On the contrary, under high temperature, the combustion of carbon only occurred on the surface, while the insufficient internal oxygen supply caused the internal C–SnO_2_ core to shrink first, forming a C–SnO_2_@SnO_2_ yolk–shell structure. Furthermore, the insufficient internal oxygen supply led the above steps to be repeated in the C–SnO_2_@SnO_2_ yolk–shell structure and produced the final double‐shell SnO_2_@SnO_2_@SnO_2_ yolk–shell structure.

In short, the soft template can be easily removed to better preserve the composition and morphology of the material, which has unique advantages. For example, for the emulsion method, the emulsion can be removed by extraction instead of calcination, which would be an excellent choice for materials with poor thermal stability. The bubble method is cost‐effective without removing template. Nevertheless, the soft template method has insufficient control over the thickness, shape, and size uniformity of the material. Here, it is suggested that researchers should pay more attention to the development of regulatory means for materials while studying the soft template method.

### 3.3 Self‐Template/Template‐Free Method

Different from the inert template materials used in the traditional hard template and soft template methods, the self‐template/template‐free method assisting in the formation of the targeted hollow structural materials without introducing inert additional templates has been developed recently. In some cases, the template material will eventually be transformed into the targeted hollow structural material or part of the targeted hollow structural material, and thus this strategy can be considered template‐free. It can not only avoid cumbersome steps but also save costs, promising its industrial application value. However, it should be pointed out that there is still great room for the self‐template/template‐free method to improve in tuning the desired shape, size, and shell thickness of the hollow structural material. According to different synthetic principles, the self‐template/template‐free method can be divided into Ostwald ripening, Kirkendall effect, surface protection etching, galvanic replacement, etc.

**Figure 5:**
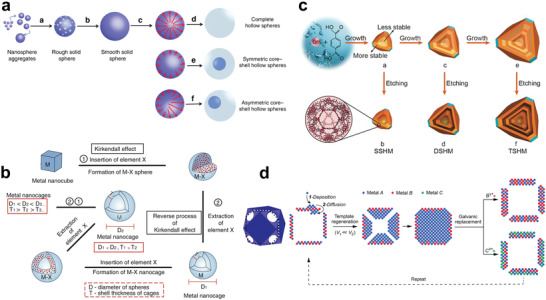
Synthesis of hollow structural materials via self‐template/template‐free method. a) Illustration of synthesis of COF hollow spheres by Ostwald ripening mechanism. Reproduced with permission.^[^ ^91^ ^]^ Copyright 2020, Springer Nature. b) Schematic diagram of the formation of hollow monometallic nanocrystals via repeated Kirkendall effect mechanism. Reproduced with permission.^[^ ^96^ ^]^ Copyright 2017, Springer Nature. c) Illustration of fabrication of hollow MIL‐101 with different shell layers via selective etching mechanism. Reproduced with permission.^[^ ^104^ ^]^ Copyright 2017, Wiley‐VCH. d) Schematic diagram of synthetic routes for highly diverse hollow nanostructures via template regeneration and galvanic replacement mechanism. Reproduced with permission.^[^ ^117^ ^]^ Copyright 2020, American Chemical Society.

Ostwald ripening is a physical phenomenon in which small particles with high surface energy are dissolved in a solvent and then deposited on larger particles to reduce the surface energy. In 2004, Yang and Zeng used Ostwald ripening for the first time to prepare hollow structural materials and synthesize hollow titanium dioxide spheres under hydrothermal conditions.^[^ ^84^ ^]^ Since then, various hollow structural materials have been synthesized based on Ostwald ripening, such as SnO_2_, TiO_2_, CuO, NiS, MoSe_2_, and covalent organic frameworks (COFs).^[^ ^85^, ^86^, ^87^, ^88^, ^89^, ^90^, ^91^ ^]^ For example, Huang and co‐workers prepared COF hollow spheres via a self‐template method based on Ostwald ripening principle (**Figure** **5**a).^[^ ^91^ ^]^ COF crystallites were first nucleated with the lowest surface energy and further assembled into large solid microspheres. After that, the surface of the crystalline microspheres gradually became smooth due to the existence of the unreacted functional groups. Eventually, the COF hollow spheres were formed through inside‐out Ostwald ripening principle. In addition, due to the different locations of Ostwald ripening, complete hollow structure, symmetrical core–shell structure, and asymmetrical core–shell structure were also produced.

The Kirkendall effect originally refers to the formation of voids due to the different diffusion rates between the diffusion of two metals at high temperatures. Alivisatos and co‐workers developed the nanoscale Kirkendall effect that can be used to synthesize hollow structural materials in 2004.^[^ ^92^ ^]^ The nanoscale Kirkendall effect describes what happens when the internal diffusion rate of solid materials is greater than the external diffusion rate of the entire materials, an internal cavity will be generated inside the structure. At present, through reasonable design, the Kirkendall effect has made great achievements in the field of hollow structural materials, such as NiO nanoparticles, Co_3_O_4_ nanotubes, and Fe_3_O_4_ nanoshells.^[^ ^93^, ^94^, ^95^ ^]^ In addition, Jin and co‐workers proposed a theoretical model that combined the positive and reverse Kirkendall effects for the first time to synthesize single‐metal Pd hollow nanocrystals.^[^ ^96^ ^]^ They found that inserting the X element with fast diffusion rate into the M nanocube could form a solid MX, and then extracting the X would produce a hollow M nanocrystal, and the process can be repeated (Figure 5b). As the number of cycles increased, the diameter (*D*) of the sphere increased, and the shell thickness (*T*) of the nanocage became thinner. On this basis, the theory was applied to experiment, phosphorus was inserted into Pd nanocube precursor through the positive process of Kirkendall effect. It was concluded that the insertion of P would disrupt the lattice and induce the transition from the Pd nanocube to the Pd–P nanosphere. The subsequent reverse Kirkendall effect process happened, during which the P was extracted by the driving force of the outward diffusion caused by the oxidation of P at high temperature. Finally, the transformation of solid nanospheres into hollow nanocages was realized.

Unlike the soft/hard template method that requires other targeted materials as the sacrificial template, selective etching utilizes itself as the sacrificial template, which is also considered to be an attractive self‐template strategy for synthesizing hollow structural materials. Selective etching requires reasonable adjustment of materials to obtain available internal unevenness. The difference in solubility between the inner and outer regions of the initial material is considered to be the key to the formation of a hollow structure. Yin and co‐workers first reported a selective etching method to prepare TiO_2_ hollow microcapsules in 2007.^[^ ^97^ ^]^ They found that the addition of poly(acrylic acid) was the key to the formation of hollow structures. It can protect the surface of TiO_2_ nanoparticles and slow down their removal in the subsequent etching process, thus effectively forming hollow structures. In the following year, they defined this method as “surface‐protected etching” and synthesized hollow SiO_2_ spheres, but introducing PVP as a surface‐protecting agent to ensure the formation of the spheres.^[^ ^98^ ^]^ In addition to poly(acrylic acid) and PVP, polymers such as sodium dodecyl sulfate, polyethylene glycol, and polyethylene imine were also considered as effective surface‐protecting agent for the construction of hollow structural materials. In addition, hollow structural materials such as Prussian Blue, Al_2_O_3_, ZnO, and CeO_2_ can also be synthesized through this way.^[^ ^99^, ^100^, ^101^ ^]^

Although surface‐protected etching does not require a presynthesized template to save costs, the added surface‐protecting agent becomes an additional cost burden. The researchers found that there is no surface‐protecting agent when manufacturing the passivation layer, and hollow structural materials can also be synthesized by etching. This is due to the nonuniformity of the self‐template material itself caused by unevenly distributed defects.^[^ ^102^, ^103^, ^104^ ^]^ An intuitive example is that solid particles prepared by the liquid phase method possess a faster growth rate in the initial growth stage, which often results in the abundant defects in the core. For example, Huo and co‐workers constructed multishelled hollow MOFs as catalysts for the first time through gradual crystal growth and subsequent selective etching (Figure 5c).^[^ ^104^ ^]^ The template of the selective etching method is itself, which serves not only as template, but also as the component of inner and outer shells, eliminating cumbersome heterogeneous coatings and making it more reproducible. However, it should be noted that it is important to avoid overetching and guarantee the accuracy of the etching.

Galvanic replacement is also an etching process, except that it requires electrode potential as the driving force. Typically, two metals with an electrochemical potential difference are selected as the anode and cathode. The metal A with a lower reduction potential is synthesized as the anode first, and then it is oxidized and dissolved when it is in electrical contact with cathode metal B with higher reduction potential. Meanwhile, metal B will be reduced and deposited on the outer surface of metal A. As the displacement reaction progresses, a hollow structural material with similar shape to metal A will eventually be constructed. In 2002, Xia and co‐workers developed the Au–Ag hollow structural material for the first time using galvanic replacement.^[^ ^105^, ^106^ ^]^ Inspired by this, various hollow structures were developed, such as nanotubes,^[^ ^107^, ^108^ ^]^ nanorods,^[^ ^109^, ^110^ ^]^ nanospheres,^[^ ^111^, ^112^ ^]^ nanocages,^[^ ^113^, ^114^ ^]^ and nanoboxes.^[^ ^115^, ^116^ ^]^ Very recently, Xia and co‐workers reported a template regenerative galvanic replacement method that can be used to synthesize high‐diversity hollow structural materials with highly adjustable parameters (Figure 5d).^[^ ^117^ ^]^ They first prepared a hollow A–B alloy nanocage by galvanic replacement, and then controlled the deposition rate to selectively grow metal A in the cavity of the hollow A–B alloy nanocage. Here, the inward diffusion rate of metal A was demanded to be much greater than the external diffusion rate to ensure that the atoms deposited on the outer surface could migrate into the cavity. The A@A–B nanostructures filled with the gaps were then used as templates for the next step of galvanic replacement. It is worth noting that this template regenerative galvanic replacement method can be repeated continuously when the nanocage has open voids, while metal A can also be replaced with alternated metal. Thus, this method breaks the stoichiometric limit of the original galvanic replacement to produce a hollow structural material with adjustable wall thickness and flexible composition.

The self‐template/template‐free method is a strategy for synthesizing hollow structural materials with simple synthetic steps, high repeatability, and large‐scale production. Compared with hard template and soft template, the self‐template/template‐free method can produce nanoscale hollow structural material without considering heterogeneous coating. Here, the development of self‐template/template‐free method including Ostwald ripening, Kirkendall effect, selective etching, and galvanic replacement is briefly reviewed. In addition, there are many other efficient methods, such as solution regeneration,^[^ ^118^, ^119^ ^]^ ion exchange,^[^ ^120^, ^121^ ^]^ and self‐assembly,^[^ ^122^, ^123^ ^]^ which are not mentioned but also have referential value. As an emerging strategy, the advantages of using the self‐template/template‐free approach will be reflected in industrial applications in the future.

## 4 Hollow Structural Materials as NRR Catalysts

Hollow structural material is a kind of nanomaterial with inner cavity, and tubular hollow nanomaterial, spherical hollow nanomaterial, and cubic hollow nanomaterial are the most common ones. In recent years, hollow structural materials are considered to be excellent catalysts, which have been vigorously studied in the fields of photocatalysis and electrocatalysis due to their large specific surface area, low density ratio, rapid mass and charge transfer, and high structural stability.

As NRR catalysts, the hollow structural materials have the following advantages. 1) The internal surfaces of the hollow structural materials are less affected by the capping agents, which are more conducive to the catalysis. At the same time, the inner cavities can effectively capture N_2_ and make N_2_ constantly collide with the inner surface, thus increasing the opportunity for the reaction site to combine with N_2_. 2) The hollow structural materials have large specific surface area in both internal and external surfaces, and abundant channels can provide large contact areas for catalytic reaction, which are beneficial for exposing more active sites and shortening mass/charge transfer pathway to further enhance the performance of NRR. 3) The self‐supported 3D geometries entitle the hollow structural catalysts with structural stability, which can effectively prevent the structural collapse during the catalytic process to obtain long‐term catalytic stability. 4) The highly unsaturated surface coordination makes the hollow structural materials have high loading capacity to disperse other active components and thus expose more N_2_ adsorption sites. The above advantages encourage researchers to develop high‐performance NRR catalysts based on hollow structural materials. In addition to hollow structural materials as intrinsic catalysts, diverse strategies have been developed by modifying and regulating the hollow structural materials in order to further enhance the catalytic activity. This section will summarize the development of hollow structural NRR catalysts from three main sections: 1) intrinsic regulation of hollow structural catalysts; 2) mesoscopic regulation of hollow structural catalysts; 3) microscopic regulation of hollow structural catalysts.

### 4.1 Intrinsic Regulation of Hollow Structural Catalysts

The hollow structural material is generally an open structure with porous shell, which helps maximize its structural advantages. Due to the existence of the cavity, the hollow material usually has large specific surface area, which would facilitate the catalytic reaction. When solid structural material is used as catalyst, the catalytic reaction only occurs on the outer surface, while when the hollow structural material acts as catalyst, the catalytic reaction occurs not only on the outer surface, but also in the pores and on the inner surface of the shell. In addition, the cavity can selectively transfer reactants, effectively limit N_2_ and reaction intermediates, and increase their collisions in the cavity, so as to improve the catalytic reaction rate in the cavity. In a word, hollow structural materials as NRR catalysts have unique advantages. This section will introduce the progress of intrinsic regulation of hollow structural catalysts.

**Figure 6:**
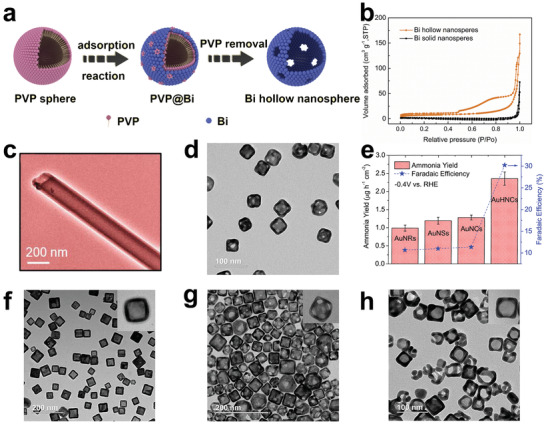
a) Schematic diagram of the synthesis of Bi hollow nanospheres. b) N_2_ adsorption/desorption isotherms of different samples. Reproduced with permission.^[^ ^130^ ^]^ Copyright 2020, Elsevier. c) TEM image of CoPc NTs. Reproduced with permission.^[^ ^137^ ^]^ Copyright 2021, American Chemical Society. d) TEM image of AuHNCs. e) NH_3_ yields and FEs of different catalysts. Reproduced with permission.^[^ ^31^ ^]^ Copyright 2018, Elsevier. f) TEM image of AuHNC‐635. g) TEM image of AuHNC‐715. h) TEM image of AuHNC‐795. Reproduced with permission.^[^ ^138^ ^]^ Copyright 2018, American Chemical Society.

As we all know, the activity of catalysts is related to their structure. For hollow catalysts, the catalytic activity can be enhanced by adjusting their morphology, shell thickness, and pore size.^[^ ^124^, ^125^, ^126^, ^127^ ^]^ According to the geometry, the hollow catalysts can be simply divided into spherical, tubular, and cubic structures. Among them, spherical hollow structural materials have been widely reported as NRR catalysts because their synthetic strategies are relatively simple. For example, Sun and co‐workers have developed VO_2_ hollow microspheres as NRR cathode materials.^[^ ^128^ ^]^ At −0.7 V versus reversible hydrogen electrode (RHE), a high NH_3_ yield of 14.85 µg h^−1^ mg^−1^_cat._ and a FE of 3.97% were obtained efficiently and stably, which were twice that of solid VO_2_ microspheres. In addition, they also synthesized hollow Bi_2_MoO_6_ spheres through a one‐step hydrothermal reaction.^[^ ^129^ ^]^ Due to the excellent structure of the catalyst, it is possible to expose more active sites and the reaction intermediate can be easily diffused. At −0.6 V versus RHE, the FE and NH_3_ yield reached 8.17% and 20.46 µg h^−1^ mg^−1^_cat._, respectively. Wang et al. reported a simple one‐pot solvent method to prepare hollow and solid Bi nanospheres (**Figure** **6**a).^[^ ^130^ ^]^ The specific surface area of hollow Bi nanospheres (23.7 m^2^ g^−1^) was nearly 16 times larger than that of solid Bi nanospheres (Figure 6b). Undoubtedly, such a high accessible surface area exposed more active sites. With the hollow Bi nanospheres as cathode materials, NRR was performed in N_2_‐saturated 0.1 m Na_2_SO_4_, and the highest NH_3_ yield was 23.4 ± 1.3 µg h^−1^ mg^−1^_cat._ at −0.4 V versus RHE, with the corresponding FE of 19.8 ± 1.1%, which was over 2 times higher than the catalytic performance of solid Bi nanospheres. Sun and co‐workers reported for the first time multishell hollow Cr_2_O_3_ microspheres as NRR catalysts with a maximum NH_3_ yield of 25.3 µg h^−1^ mg^−1^_cat._ and the corresponding FE was 6.78% at −0.9 V versus RHE.^[^ ^131^ ^]^

Proton hydrogenation is also an important step in determining the reaction kinetics during the NRR reaction, especially for proton‐deficient alkaline electrolytes. Previous studies have shown that the phosphate in metal phosphate can be used as a proton transport carrier to promote proton transfer to the active site.^[^ ^132^ ^]^ In addition, metal–N_2_ battery, as a kind of dual function secondary battery combining energy storage and artificial nitrogen fixation, has attracted extensive attention recently.^[^ ^133^, ^134^ ^]^ In view of this, Yuan and co‐workers derived hollow molybdenum phosphate microsphere (MoPi/HSNPC) by pyrolyzing molybdenum phosphate as a dual‐function catalyst for Al–N_2_ batteries.^[^ ^135^ ^]^ The MoPi/HSNPC achieved the highest NH_3_ yield of 18.66 µg h^−1^ mg^−1^_cat._ and a FE of 9.04% in NRR test. During the discharge of the Al–N_2_ battery, an NH_3_ yield of 13.47 µg h^−1^ mg^−1^_cat._ and a FE of 5.06% were also obtained. A series of control experiments showed that the excellent catalytic performance came from the hollow structure that promoted the diffusion of reactants and exposed more active sites, as well as the promotion of proton transfer by phosphate. In the same year, they developed hollow cobalt phosphate microsphere (CoPi/HSNPC) as a dual‐function catalyst for Zn–N_2_ batteries.^[^ ^136^ ^]^ Due to the enhanced proton conduction and numerous active sites, the CoPi/HSNPC obtained an NH_3_ yield of 16.48 µg h^−1^ mg^−1^_cat._ and a high FE of 4.46%, and the FE can be further increased to 24.42% when used as a Zn–N_2_ battery. These studies offer guiding significance for the development of other metal phosphate catalysts, and provide new insights into the design of nitrogen fixation devices under mild conditions.

In addition to spherical hollow structural catalysts, tubular structural catalysts with hollow cylindrical structures have also been developed as NRR catalysts. They not only have the advantages of various hollow structural materials, but also provide fast mass transfer channels. Most recently, *β*‐cobalt phthalocyanine nanotubes (CoPc NTs) were developed on a large scale by Ghorai et al. to improve the activity and selectivity for NRR (Figure 6c).^[^ ^137^ ^]^ The tubular hollow structural material not only provided a high specific surface area (41.5 m^2^ g^−1^) to expose abundant active sites, but also served as a mass transfer channel for NRR. Studies have found that nonmetallic phthalocyanine can effectively reduce the hydrogen adsorption capacity of transition metal Co. The NRR test was carried out in 0.1 M HCl electrolyte, and the prepared CoPc NTs obtained an ultrahigh NH_3_ yield of 107.9 µg h^−1^ mg^−1^_cat._ and an excellent FE of 27.7% at −0.3 V versus RHE. The ^15^N_2_ isotope tracer experiments have confirmed that the NH_3_ production was the result of N_2_ reduction rather than the contamination caused by the N‐contained in the material itself, which was very convincing characterization for the N‐containing catalysts. Notably, CoPc NTs had bon electrocatalytic selectivity as well as structural stability, which were also important criteria to evaluate excellent catalysts. The density functional theory (DFT) calculation results indicated that cobalt with a low d‐band center value was the main active site of catalysis, and associative alternating pathway was the preferred pathway for CoPc NTs. In conclusion, the effective inhibition of the hydrogen adsorption of transition metals by nonmetallic phthalocyanines, the enhancement of mass transfer by the tubular hollow structure, and the activation of N≡N by the d orbital of Co are considered to be key factors for the excellent performance. These results provide guidance for the further development and design of phthalocyanine catalysts based on other transition metals.

Compared with other hollow structural materials, cubic hollow structural material with sharp edges has a larger specific surface area, as shorter ion transport path, and more unsaturated surface atoms, which is considered to be a more effective catalyst. El‐Sayed and co‐workers^[^ ^31^ ^]^ prepared hollow porous gold nanocages (AuHNCs) using silver nanocubes as templates by the galvanic replacement method (Figure 6d). AuHNCs exhibited an excellent FE of up to 30.2% in the 0.5 m LiClO_4_ electrolyte at −0.4 V versus RHE, and the highest NH_3_ yield of 3.98 µg cm^−2^ h^−1^ was achieved at −0.5 V versus RHE. At a more negative potential, the active sites were more likely to be occupied by hydrogen than nitrogen due to the influence of competitive HER, leading to a significant drop in the FE and NH_3_ yields. After five consecutive cycles, the catalytic performance of the AuHNCs was maintained at about 93.8%, which demonstrated the durability of the AuHNCs. Noteworthy, the NH_3_ yields for Au nanospheres (diameter 35 nm) and Au nanocubes (AuNCs, side length 35 nm) were slightly increased than that for Au nanorods (length 42 nm and width 12 nm), as shown in Figure 6e. This result indicates that materials with sharper edges have more unsaturated surface atoms, which can provide more active sites than those with smooth surfaces. In addition, the enhanced catalytic performance of AuHNCs over AuNCs is attributed to the fact that N_2_ molecules can be trapped by their cavities, which would extend the residence time of N_2_ molecules on the inner surface of the catalyst, thus facilitating the conversion of N_2_ to NH_3_.

The limitation of the cavity on reactants enables the hollow structural material to possess enhanced selectivity, and therefore, reasonable design of the pore size is very important. For pore with extreme small size, it is difficult for reactants to enter into the cavity, while for pore with large pore size, it is difficult for the cavity to maintain the restriction effect on the reactants. Nazemi and El‐Sayed explored the influence of the pore size of hollow structural materials on the performance of NRR.^[^ ^138^ ^]^ AuHNCs (AuHNC‐635, AuHNC‐715, AuHNC‐795) with different peak localized surface plasmon resonances (LSPRs) were prepared by adding different amounts of Au^3+^ to replace the Ag nanocubes (Figure 6f–h). They found that as the LSPR became larger, the pore size of the nanocage increased. It is worth noting that a further increase in the pore size of the catalysts reduced the catalytic activity, due to the excessive reduction of the active surface area and the weakening of restricted ability of reactants. Compared with AuHNC‐635 and AuHNC‐795, AuHNC‐715 displayed the best catalytic performance with NH_3_ yield of 3.74 µg h^−1^ cm^−2^ and FE of 35.9%. Hence, the design of the pore size of the hollow catalysts should conform to the characteristics to guarantee the diffusion of reactants and products without excessive loss of surface area.

To sum up, hollow structural materials are promising NRR catalysts due to their advantages of large specific surface area, rapid mass transfer, and restriction of reactants. In order to obtain better catalytic performance, pore size and morphology of the hollow structural material should be optimized. In addition, although there has not been a detailed study on the impact of the shell thickness of hollow structural materials on the catalytic performance of NRR, it is conceivable that the shell thickness would have a certain impact on the exposure of catalytic active sites. At the same time, it is also worth exploring that the modified or hybrid hollow structural materials possess stronger roughness and can expose more N_2_ adsorption sites than smooth hollow structural materials. Furthermore, some complex multishell hollow structural materials with enhanced reactant confinement capabilities are worth further exploration, which may further improve the catalytic performance of NRR.

### 4.2 Mesoscopic Regulation of Hollow Structural Catalysts

Although the intrinsic hollow structural catalysts have various considerable advantages, the rational design of composite materials constructed with hollow structural materials as substrates may result in efficient catalysts with enhanced performance. Hollow structural materials with large specific area and high stability are suitable carriers to load other active ingredients efficiently and stably by the postsynthetic means or the impregnation method. The large specific surface area allows the hollow structural material to load more active ingredients, thereby upgrading N_2_ adsorption sites. The high structural stability enables the hollow structural materials to prevent the structure from being collapsed or destroyed when loading other active ingredients, and effectively averts the agglomeration of active sites. Meanwhile, the excellent electrolyte diffusion channel provided by hollow structural materials can also bring better N_2_ accessibility to the active components. Moreover, the adjustment of the interfacial electrons and the enhanced electron transfer between different components can further optimize the catalytic activity of NRR. Therefore, it is an effective method to further optimize the hollow structural catalysts under the mesoscopic level.

The term “mesoscopic” refers to the intermediate state between “macroscopic” and “microscopic,” and is applied in microstructures with the characteristics of crossover and intersection between quantum state and classical state. Therefore, mesoscopic materials endow nanomolecules and nanomaterials with specific properties, providing broad applications. Commonly, the mesoscopic materials refer to nanomaterials that have at least 1D between 1 and 100 nm on the 3D scale, and they are often used as building blocks to participate in the formation of materials. In the past few decades, studies have found that metallic materials with nanoscale dimensions belong to mesoscopic materials. In this state, metallic nanomaterials possess unique localized surface plasmon (LSP) effects, small size effects, and surface effects.^[^ ^139^, ^140^ ^]^ LSP effects play a key role in enhancing the electric field on the surface of nanoparticles, small size effects play role in increasing the specific surface area, and the surface effects play role in altering surface energy and surface binding energy of nanoparticles. Combining these unique properties, mesoscopic materials as electrocatalysts exhibit excellent NRR performance. According to different regulation methods, the mesoscopic regulation of hollow structural materials is simply divided into two categories: hollow structural materials as carriers to load nanomaterials and hollow structural materials as substrates to construct heterostructures by coupling with other components.

#### 4.2.1 Loading Nanomaterials

The catalytic activity of electrocatalysts is related to the size of the nanomaterials. The smaller the size, the larger proportion of surface active atoms the nanomaterials will have, which makes nanoscale materials to have potential as catalysts. However, nanomaterials with extreme small size commonly possess high surface‐unsaturated coordination and enhanced surface energy, which would result in uncontrolled spontaneous agglomeration. Therefore, it is necessary to find a suitable carrier to anchor the highly active nanomaterials to prevent their agglomeration.

The hollow structural material is considered to be a good carrier due to its large specific surface area and high surface‐unsaturated coordination, which can support and disperse various nanoparticles, such as nanoparticles, molecules, and quantum dots. On this basis, tubular hollow structural materials with continuous interconnecting ion diffusion paths and enhanced permeability have attracted much attention. Carbon nanotubes (CNTs), as a typical tubular hollow structural material, have been widely used in the fields of energy storage and energy conversion.^[^ ^141^, ^142^ ^]^ Although CNTs have a very low catalytic activity (≈1 µg h^−1^ mg^−1^_cat._) during electrocatalytic reactions,^[^ ^143^ ^]^ their excellent conductivity, large specific surface area, and high surface unsaturated coordination make them excellent catalyst carriers, which could load and disperse the nanoparticles uniformly and stably. Ding and co‐workers reported iron phthalocyanine/oxidized multiwalled carbon nanotubes (FePc/O‐MWCNT) as NRR catalysts by dispersing FePc on O‐MWCNT.^[^ ^144^ ^]^ At −0.3 V versus RHE, FePc/O‐MWCNT exhibited a FE of 9.73% and an NH_3_ yield of 36 µg h^−1^ mg^−1^_cat._, which were better than pure FePc and O‐MWCNT. Fe_3_C also could be considered as an effective NRR catalyst. Huang and co‐workers prepared N‐doped carbon nanotube/Fe_3_C nanoparticle through a simple pyrolysis method.^[^ ^145^ ^]^ The results show that: 1) CNTs could effectively increase the specific surface area of the catalyst; 2) Fe_3_C acted as the active center of the NRR catalyst; 3) the high content of pyridine nitrogen not only contributed to N_2_ adsorption, but also improved the hydrophilicity of the catalyst. Li et al. used MWCNT as a carrier to anchor CuCo_2_S_4_ and prepared CuCo_2_S_4_/MWCNT as a highly efficient NRR catalyst.^[^ ^146^ ^]^ CuCo_2_S_4_ nanoparticles were known to easily self‐agglomerate without carriers to form particles ranging from 200 to 300 nm in size. The introduction of MWCNT effectively changed the dispersion of CuCo_2_S_4_ to a particle size of 30–50 nm, which exposed more active sites. In addition, the introduction of MWCNT also increased the specific surface area of the catalyst and improved the electron transfer capacity. CuCo_2_S_4_/MWCNT obtained a high NH_3_ yield of 137.5 µg h^−1^ mg^−1^_cat._ and a corresponding FE of 8.7% in 0.1 m Na_2_SO_4_ electrolyte.

**Figure 7:**
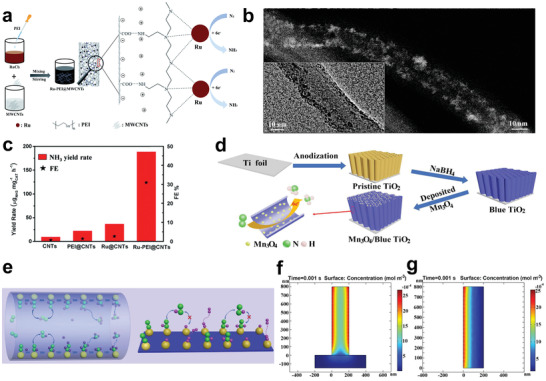
a) Schematic illustration of the synthetic process of Ru–PEI@MWCNTs. b) HRTEM image of Ru–PEI@MWCNTs. c) NH_3_ yields and FEs of different catalysts. Reproduced with permission.^[^ ^147^ ^]^ Copyright 2019, Royal Society of Chemistry. d) Schematic illustration of the fabrication of Mn_3_O_4_/b‐TiO_2_ nanotube arrays. e) Schematic of reaction intermediates confined in nanotube and planar models. f) Concentration of *N_2_ intermediates for nanotube model. g) Concentration of *N_2_ intermediates for planar model. Reproduced with permission.^[^ ^148^ ^]^ Copyright 2020, Wiley‐VCH.

Ru catalyst is considered structure‐sensitive for NRR by DFT because Ru metal could increase the binding strength of N_2_ and lower the energy barrier for N_2_ detachment. It is effective to seek a suitable substrate for Ru dispersion to improve the atomic utilization. Recently, Ma and co‐workers assembled ruthenium polyethyleneimine (Ru–PEI) onto carboxyl‐modified CNTs by electrostatic force and prepared Ru–PEI@MWCNTs as an efficient NRR catalyst (**Figure** **7**a).^[^ ^147^ ^]^ No aggregated particles were found by scanning electron microscopy (SEM) and transmission electron microscopy (TEM), which confirmed that MWCNTs could uniformly disperse Ru–PEI. As shown in Figure 7b, a large number of bright spots were found in the high‐resolution TEM (HRTEM) image, which further confirmed the uniform loading of Ru–PEI. Further research found that PEI with abundant NH_2_ groups had strong coordination ability, which was key to the stable and efficient loading of Ru–PEI on MWCNTs. It was difficult to load substantial Ru on MWCNTs without introducing PEI. As shown in Figure 7c, the carefully designed Ru–PEI@MWCNTs exhibited a high ammonia yield of 188.9 µg h^−1^ mg^−1^_cat._ and an excellent FE of 30.93%, which were better than other comparative samples.

Although CNTs have many advantages as substrates, their low NRR catalytic activity hinders the efficient production of NH_3_. Therefore, the development of active hollow substrate is particularly important. Wu and co‐workers reported the composite Mn_3_O_4_/blue titanium dioxide (b‐TiO_2_) as an efficient NRR catalyst, which was prepared by depositing Mn_3_O_4_ nanoparticles on b‐TiO_2_ nanotubes (Figure 7d).^[^ ^148^ ^]^ It was found that different loading amounts exerted an impact on the catalytic activity of the catalysts. When the loading capacity of Mn_3_O_4_ was 0.57 mg cm^−2^ (0.57Mn_3_O_4_/b‐TiO_2_), the catalyst exhibited the best NRR performance with NH_3_ yield of 1.61 × 10^−10^ mol s^−1^ cm^−2^ at −0.45 V versus RHE. As the loading amount of Mn_3_O_4_ further increased, excessive Mn_3_O_4_ aggregated and caused blockage of TiO_2_ nanotubes, resulting in a decrease in specific surface area, thereby reducing the catalytic performance. In addition, the excellent performance of 0.57Mn_3_O_4_/b‐TiO_2_ came from not only the simple superposition of 0.57Mn_3_O_4_ and b‐TiO_2_, but also the limiting effect of nanotube cavity on the intermediate, thereby effectively improving the NRR reaction kinetics (Figure 7e). As shown in Figure 7f,g, finite element analysis was used to simulate the distribution of intermediate concentration in nanotube and planar mode. The results showed that nanotube can effectively limit the reaction intermediates and increase the local intermediate concentration, while the plane is difficult to limit the intermediates.

Black phosphorus has attracted wide attention due to its excellent electrochemical properties, among which the quantized one shows good catalytic activity. However, pure black phosphorous quantum dots (BP QDs) will inevitably undergo agglomeration, which will cause the loss of active sites. For this reason, Ding and co‐workers successfully self‐assembled BP QDs onto tin dioxide nanotubes reduced by sodium borohydride (BP@SnO_2−_ *_x_*) through tin–phosphorus coordination interactions.^[^ ^149^ ^]^ At −0.4 V versus RHE, BP@SnO_2−_ *_x_* showed the highest NH_3_ yield of 48.87 µg h^−1^ mg^−1^_cat._ and a FE of 14.6%, which were better than individual BP QDs (29.22 µg h^−1^ mg^−1^_cat._) and SnO_2−_ *_x_* (8.64 µg h^−1^ mg^−1^_cat._), respectively. In addition, after a long‐term electrolytic experiment, BP QDs were still stably loaded on SnO_2−_ *_x_* without agglomeration, indicating that BP@SnO_2−_ *_x_* had excellent stability.

#### 4.2.2 Construction of Heterostructures

Heterojunction refers to the junction or interface between different materials, that is, the crystal interface formed by the combination of two materials with different bandgaps. Therefore, heterostructure is defined as a structure with more than two layers containing a heterojunction. Compared with a single component, the interface in heterostructure is conducive to increasing active sites and electronic regulation, thereby playing an important role in improving the catalytic performance.^[^ ^150^, ^151^ ^]^ Meanwhile, the heterostructure can speed up the electron transfer due to electronegative differences between dissimilar components, further facilitating the adsorption/desorption of reaction intermediates.^[^ ^152^, ^153^ ^]^ In addition, different components can play separate roles, thereby synergistically optimizing catalytic performance.^[^ ^154^, ^155^ ^]^ Among them, the hollow structural material can be used as a carrier loading other nanomaterials to construct a heterostructure catalyst, which can achieve rapid mass diffusion and effectively reduce the overpotential.^[^ ^156^, ^157^ ^]^ Furthermore, the structural stability of the hollow structural material can reduce the agglomeration of active materials during the reaction and maximize the exposure of active sites.^[^ ^158^, ^159^, ^160^ ^]^

**Figure 8:**
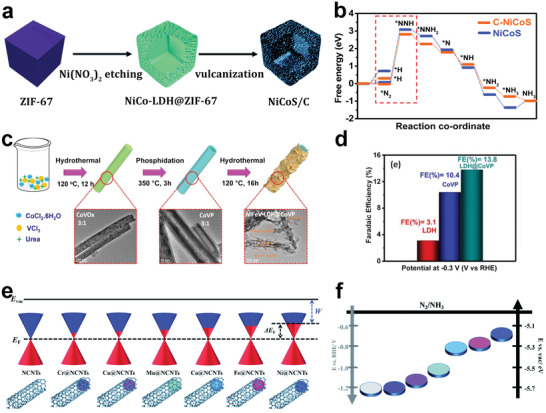
a) Schematic illustration of the formation process of NiCoS/C nanocage. b) Free energy diagram of N_2_ reduction on NiCoS/C and NiCoS. Reproduced with permission.^[^ ^161^ ^]^ Copyright 2020, Royal Society of Chemistry. c) Schematic diagram for the synthetic procedure of CoVP@NiFeV–LDHs. d) FEs for NiFeV–LDHs, CoVP, and CoVP@NiFeV–LDHs. Reproduced with permission.^[^ ^162^ ^]^ Copyright 2020, Elsevier. e) Schematic illustration of the design concept of work function of M@NCNTs. *E*_vac_ and *E*_F_ represent vacuum level and Fermi level, respectively. f) The corresponding energy level diagram. Reproduced with permission.^[^ ^163^ ^]^ Copyright 2020, Royal Society of Chemistry.

Wang and co‐workers reported NiCoS/C nanocage, where NiCoS and C possessed strong chemical coupling effect.^[^ ^161^ ^]^ This nanocage was obtained by vulcanizing NiCo–layered double hydroxide (LDH)@ZIF‐67, exhibiting a high NH_3_ yield of 58.5 µg h^−1^ mg^−1^_cat._ and a FE of 12.9% at 0.1 m Li_2_SO_4_ (**Figure** **8**a). The nitrogen temperature‐programmed desorption (N_2_‐TPD) confirmed that NiCoS/C nanocage exhibited enhanced N_2_ adsorption capacity compared with a single NiCoS nanocage prepared by prolonging the vulcanization time. In addition, the NiCoS/C nanocage was prior to NiCoS in terms of structural stability and HER inhibition ability. The DFT calculations further confirmed that the coupling interaction between NiCoS and C could reduce the free energy barrier in the NRR rate‐determining step (Figure 8b). This research provides new ideas for the design and development of polymetallic hollow heterostructural materials as NRR catalysts.

Electrocatalytic NRR is considered to be a PCET reaction. In this reaction, the adsorption of N_2_ molecules on the active site and the subsequent activation by accepting electrons are believed to be important steps. Since the complete reduction of N_2_ to NH_3_ requires N_2_ activation and 6‐electron PCET reaction, good electrical conductivity and fast electron transfer are also important criteria for evaluating catalysts. The heterostructures play a unique role in accelerating interfacial charge transfer and enhancing electrical conductivity. Typical LDH nanosheets possessing large surface areas can expose more active sites, but their electrical conductivity is relatively poor. To address this issue, Yan and co‐workers reported a heterogeneous structure CoVP@NiFeV–LDHs with accelerated interfacial charge transfer and enhanced conductivity by loading NiFeV–LDHs on CoVP hollow nanotubes (Figure 8c).^[^ ^162^ ^]^ Electrochemical impedance spectroscopy analysis verified that the heterostructure of CoVP@NiFeV–LDHs had the best electron transport capability relative to single‐component CoVP and NiFeV–LDHs. The hollow CoVP nanotubes not only enhanced the diffusion kinetics of NRR, but also provided structural stability for the dispersion of NiFeV–LDHs. In the 0.05 m H_2_SO_4_ electrolyte, the finely designed CoVP@NiFeV–LDHs obtained the highest NH_3_ yield of 1.6 × 10^−6^ mol h^−1^ cm^−2^ at −0.3 V versus RHE. As shown in Figure 8d, CoVP@NiFeV–LDHs demonstrated the highest FE (13.8%) relative to CoVP (10.4%) and NiFeV–LDHs (3.1%). In addition, CoVP@NiFeV–LDHs also had good stability, as evidenced by no obvious performance degradation after 8 consecutive cycles.

Recently, Zhang and co‐workers designed a series of metal‐encapsulated N–CNT Schottky heterojunctions as NRR catalysts, and systematically studied the regulation of work function on catalytic activity.^[^ ^163^ ^]^ As shown in Figure 8e, the work functions of M@NCNTs decreased in the order of metal M being Cr, Cu, Mn, Co, Fe, Ni, with NCNTs having the highest work function and Ni@NCNTs having the lowest work function. As shown in Figure 8f, the energy required to drive the NRR reaction dropped as the work function decreased. Ni@NCNT was therefore considered as the most promising and high‐efficient NRR catalyst. The correction of the charge density of this Mott–Schottky heterostructure was further proved by comparing the peak shift of the pyridine nitrogen in the X‐ray photoelectron spectroscopy (XPS) spectrum. The finely designed Ni@NCNTs obtained the highest NH_3_ yield of 53.88 µg h^−1^ mg^−1^_cat._ and the maximum FE of 7.33% at −0.5 and −0.3 V versus RHE, respectively. All in all, the decrease in work function can increase the charge density near the Fermi level, thereby accelerating electron transfer to promote the activation of N_2_. The above studies show that researchers should pay attention to the electron‐supply capacity of catalysts while improving their activity.

**Figure 9:**
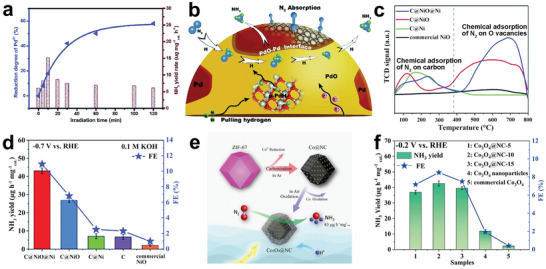
a) Reduction degree and NH_3_ yield rates of PdO/CNTs with different irradiation times. b) Alternative hydriding pathway for NRR on the PdO–Pd interface. Reproduced with permission.^[^ ^164^ ^]^ Copyright 2019, Royal Society of Chemistry. c) N_2_‐TPD profiles of different catalysts. d) NH_3_ yield rates and FEs of different catalysts at −0.7 V versus RHE. Reproduced with permission.^[^ ^165^ ^]^ Copyright 2020, Royal Society of Chemistry. e) Schematic illustration of the preparation of core–shell Co_3_O_4_@NC. f) NH_3_ yield rates and FEs of different catalysts at −0.2 V versus RHE. Reproduced with permission.^[^ ^166^ ^]^ Copyright 2019, American Chemical Society.

In addition to improving the conductivity of the catalyst and accelerating electron transfer, the construction of heterostructures can also form a unique heterogeneous interface to promote the adsorption of reactants and finally improve the catalytic activity. Among them, the metal–metal oxide interface is considered to be the best choice for improving the catalytic activity due to the strong interfacial interaction force between them. By adjusting the laser irradiation time, Liang and co‐workers partially reduced the PdO nanoparticles grown on CNTs to Pd, forming PdO/Pd heterojunction CNTs (PdO/Pd/CNTs).^[^ ^164^ ^]^ As shown in **Figure** **9**a, the irradiation time determined the reduction degree of Pd^2+^, which directly affected the NRR performance. Among them, the sample irradiated for 10 min showed the highest NH_3_ yield (18.2 µg h^−1^ mg^−1^_cat._). As shown in Figure 9b, when N_2_ reached the PdO/Pd heterogeneous interface, Pd would capture N_2_ to form Pd—N bond, while adjacent PdO would form *α*‐PdH with activated protons, shortening the subsequent proton transfer distance and effectively reducing the overpotential of NRR. Coincidentally, Luo et al. used Ni‐based MOF to derive C@NiO@Ni microtubes by adjusting the annealing method as an efficient NRR catalyst under alkaline conditions, showing high NH_3_ yield (43.15 µg h^−1^ mg^−1^_cat._) and FE (10.9%).^[^ ^165^ ^]^ Both electron paramagnetic resonance (EPR) and XPS spectra confirmed the rich oxygen vacancies in C@NiO@Ni and C@NiO. As shown in Figure 9c, N_2_‐TPD demonstrated that oxygen vacancy could enhance the chemical adsorption capacity of N_2_, indicating that oxygen vacancy was the active site of the catalyst. Although oxygen vacancies existed, the abundant NiO/Ni interface in alkaline electrolyte was proven to be more critical for proton capture to further promote the NRR reaction. As shown in Figure 9d, C@NiO@Ni exhibited NH_3_ yield that was 0.62 times higher than C@NiO, which was much higher than C@Ni, C, and commercial NiO. However, the oxygen vacancy concentration of C@NiO@Ni is only 0.23 times higher than the oxygen vacancy concentration of C@NiO. The performance difference was probably due to the influence of the NiO/Ni interface. This work develops complex catalytic systems and emphasizes the importance of detailed characterization of the contribution of each component, which guides researchers to explore multifunctional electrocatalysis.

Core–shell structure is also a typical heterostructure, which has the advantages of adjustable size, large specific surface area, and unique core–shell interface. In some cases, a stable outer shell can act as a protective layer to protect the inner core from erosion. On this basis, the core–shell structure with hollow characteristics enables the fast capture of reactants. Wang and co‐workers prepared Au_3_Cu@Cu nanocages with a hollow porous core–shell structure using Cu_2_O as a template, which demonstrated a high NH_3_ yield of 33.97 µg h^−1^ mg^−1^_cat._ and an excellent FE of 21.41% at 0.1 m Na_2_SO_4_.^[^ ^167^ ^]^ The hollow porous heterostructure effectively increased the specific surface area while limiting the reactant to increase the contact frequency between the active site and the reactant. At the same time, the outer layer of Cu readily inhibited competitive HER, which would improve the NRR selectivity. Recently, Luo et al. developed a N‐doped carbon/Co_3_O_4_ (Co_3_O_4_@NC) core–shell structure derived from ZIF‐67 for the first time, as shown in Figure 9e.^[^ ^166^ ^]^ The materials with different morphologies (Co_3_O_4_@NC‐5, Co_3_O_4_@NC‐10, Co_3_O_4_@NC‐15) were prepared at different calcination times (5, 10, 15 h) under air atmosphere due to different migration rates. As the oxidation time increased, the degree of graphitization and specific surface area of Co_3_O_4_@NC increased. In this work, oxygen vacancies were also considered to be a key factor in determining catalytic performance, because they can inject captured metastable electrons into the antibonding orbital of N_2_ to weaken N≡N. Finally, the core–shell structure Co_3_O_4_@NC‐10 with the highest oxygen vacancies obtained the best NRR performance (NH_3_ yield: 42.58 µg h^−1^ mg^−1^_cat._, FE: 8.5%) compared to its counterpart and Co_3_O_4_ nanoparticles, as shown in Figure 9f. This work encourages researchers to combine multiple advantages into one catalyst, which may play an unexpected role in synergistically promoting NRR. It is equally important that this synergistic effect needs to be explored in depth to clarify their respective contributions.

In short, the fine edge exposed part of heterostructures can provide more adsorption sites for N_2_. In terms of structures, the hollow heterostructures have both the advantages of enhanced conductivity of the heterojunctions and rapid mass diffusion of hollow structures. In terms of materials, heterostructural materials can integrate the advantages of different components, thereby optimizing catalytic performance. It is worth noting that the excellent catalytic performance of heterostructural materials is often attributed to the synergy between different components. However, this synergy needs to be further investigated in detail to clarify their internal mechanism.

### 4.3 Microscopic Regulation of Hollow Structural Catalysts

In addition to mesoscopic regulation methods, the catalytic performance of hollow structural catalysts can be further improved through microscopic regulation strategies. Here, microscopic regulation is defined as the control of at least 1D size of materials in the 3D space on the atomic scale in the range of 0.1–1 nm. Compared with mesoscopic regulation, microscopic regulation is more inclined to atomic‐level control to enhance the inherent activity of the catalysts. The hollow structural catalysts can be modified in favor of NRR through various microscopic regulation strategies. For example, after microscopic regulation, the local coordination environment of hollow structural materials will be adjusted to improve the affinity between the catalyst and N_2_. Meanwhile, the electronic structure, electron transmission, and conductivity can be optimized and surface defects can be created through microscopic regulation. In conclusion, microscopic regulation strategies render the hollow structural catalysts more excellent catalytic performance. Considering the different regulation methods, the microscopic regulation of hollow structural materials is simply divided into the following categories: doping engineering, vacancy engineering, single atom engineering, etc.

#### 4.3.1 Doping Engineering

The heteroatom doping is an effective strategy to regulate the physical or chemical properties of materials because heteroatoms and intrinsic atoms have different electronic structures. The introduction of heteroatoms will create a special coordination environment on the surface of the materials, adjust the electronic structure, and manufacture defects, which will help increase the active sites and improve the binding ability with N_2_. Therefore, heteroatom doping is considered as an effective method to improve catalytic activity and has been widely studied in the field of electrocatalytic NRR. For example, Sun and co‐workers synthesized Fe‐doped TiO_2_ as a NRR catalyst, which showed 5 times higher NH_3_ yield than undoped TiO_2_.^[^ ^168^ ^]^ The introduction of iron spontaneously increased the oxygen vacancy content in the TiO_2_, which was thought to be the active site that promoted N_2_ activation. Zhang and co‐workers designed a dual‐function catalyst Fe‐doped SnO_2_, which exhibited very excellent catalytic performance compared to undoped SnO_2_.^[^ ^169^ ^]^ Experiments and DFT calculations indicated that a part of the introduced heteroatom Fe was doped into the crystal lattice to improve the conductivity of SnO_2_, and other parts were anchored in the oxygen vacancy to form single‐atom Fe to enhance the adsorption and activation of N_2_. Quan and co‐workers prepared N‐doped porous carbon as NRR cathode material by pyrolysis of ZIF‐8.^[^ ^170^ ^]^ Studies have found that N doping adjusted the electronic structure of the material and induced charge polarization, in which pyridinic N and pyrrolic N were the active sites for N_2_ adsorption and activation.

As an excellent strategy, heteroatom doping has also been applied to further enhance the catalytic activity of hollow carbon materials. The heteroatom doping of carbon‐based hollow structural materials can create defects, adjust the electronic structure, and increase the number of active sites. Sun and co‐workers prepared S‐doped carbon nanospheres (S–CNSs) as an excellent NRR catalyst by hydrothermal treatment.^[^ ^171^ ^]^ Raman spectra showed that S–CNSs had higher disorder degree and more defects than undoped carbon nanospheres (CNSs). N_2_‐TPD revealed that S–CNSs had stronger chemical and physical adsorption capacity for N_2_. Also, S–CNS achieved a high NH_3_ yield of 19.07 µg h^−1^ mg^−1^_cat._, which was more than 5 times higher than CNS. Recently, Hu and co‐workers synthesized P‐doped carbon nanotubes (P–CNTs) as NRR electrocatalyst through simple grinding and temperature‐assisted phosphorization.^[^ ^172^ ^]^ In 0.25 m LiClO_4_, the P–CNTs showed a high NH_3_ yield of 24.4 µg h^−1^ mg^−1^_cat._ at −1.1 V versus RHE and a FE of 12.5% at −0.3 V versus RHE. Raman spectroscopy analysis found that P–CNTs had a higher *I*_D_/*I*_G_ intensity ratio compared with the original CNTs, indicating that more defects were generated on the surface of P–CNTs. Control experiments found a linear relationship between NH_3_ production and P doping, revealing that the P site was the true active center of NRR. The results of the Mulliken charge density redistribution showed that the heteroatom P contributed to the formation of Lewis acid sites to adsorb N_2_ on the weak Lewis base due to its high positive charge density, which was an intuitive evidence that the P site was the reaction center. The free energy of N_2_ adsorption on P atoms (−0.24 eV) was better than that on C atoms (−0.13 eV), further confirming that P was the active site of NRR. Eventually, the reaction followed an associative distal pathway which was demonstrated by UV and in situ infrared spectroscopy.

**Figure 10:**
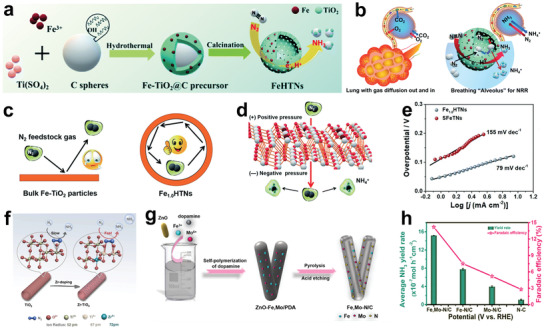
a) Schematic diagram of the synthesis of FeHTN. b) Left: scheme of a human lung and a single alveolus. Right: schematic illustration of an artificial breathing “alveolus” of FeHTNs for NRR. c) Schematic of the collision behaviors of N_2_ on the surfaces of different samples. d) Schematic of pressure differences inside and outside the cavity of Fe_1.0_HTN. e) Tafel plots of different samples for NRR. Reproduced with permission.^[^ ^174^ ^]^ Copyright 2021, Royal Society of Chemistry. f) Schematic illustration of formation of adjacent bi‐Ti^3+^ sites and oxygen vacancies owing to Zr doping in anatase TiO_2_. Reproduced with permission.^[^ ^175^ ^]^ Copyright 2019, Springer Nature. g) Scheme illustration of the synthetic process of Fe,Mo–N/C. h) NH_3_ yields and FEs of different catalysts. Reproduced with permission.^[^ ^176^ ^]^ Copyright 2020, American Chemical Society.

Metal‐based hollow structural materials doped with heteroatoms have also been developed as electrocatalysts for NRR. The heteroatom doping of metal‐based hollow structural materials can change the electronic structure and introduce vacancies, leading to enhanced reaction intermediates adsorption and rapid charge transfer.^[^ ^173^ ^]^ As shown in **Figure** **10**a, Yuan and co‐workers prepared Fe‐doped titanium dioxide hollow nanospheres (FeHTNs) by a hard template method.^[^ ^174^ ^]^ HRTEM indicated that many oxygen vacancies existed in FeHTNs due to the boosting of Fe doping. XPS spectroscopy and electron spin resonance both confirmed that Fe doping can increase the oxygen vacancy content of TiO_2_. FeHTNs with addition amount of 1.0 wt% Fe (Fe_1.0_HTNs) exhibited a significantly enhanced NH_3_ yield (43.14 µg h^−1^ mg^−1^_cat._), which was higher than that of undoped TiO_2_ hollow nanospheres and solid Fe‐doped TiO_2_ nanoparticles (SFeTNs). Such excellent performance was attributed not only to the increase of oxygen vacancy content by Fe doping, but also to the hollow structural cavity that promoted N_2_ absorption. As shown in Figure 10b, they proposed a concept of “alveolus,” similar to the breathing process of mammalian alveoli. Theoretical calculations showed that the number of N_2_ collision on the surface of Fe–TiO_2_ nanoparticles may be one, but the probability of N_2_ collisions in the FeHTNs cavity would be multiple until NH_3_ was formed (Figure 10c). Meanwhile, the discrepancy in the solubility of NH_3_ causes the pressure difference between inside and outside the cavity to drive N_2_ into the cavity (Figure 10d). In addition, compared to SFeTNs, Fe_1.0_HTNs had a higher Tafel slope value, which further confirmed the improvement of NRR kinetics due to the existence of the hollow structure (Figure 10e). The NRR performances of Fe_1.0_HTNs with different mechanically crushed times were compared. As the crushing time increased, the specific surface area of the material increased, but the catalytic performance continued to decline, which further confirmed the significance of the cavity limiting effect to improve NRR properties.

Recently, Zheng and co‐workers discovered that the adjacent bi‐Ti^3+^ pairs formed on anatase TiO_2_ were key sites for N_2_ adsorption and activation using DFT calculations.^[^ ^175^ ^]^ As shown in Figure 10f, Zr–TiO_2_ with the contiguous bi‐Ti^3+^ pairs and oxygen vacancies was formed by doping the same valance dopant Zr^4+^ ions on anatase type TiO_2_ nanotubes. In addition, Ce^4+^ with a larger ion radius could also be utilized as dopant (Ce–TiO_2_) to produce oxygen vacancies, but it would be reduced to Ce^3+^ during the process of creating oxygen vacancies and may even destroy the crystal structure of TiO_2_. The optimal NH_3_ yield (8.90 µg h^−1^ cm^−2^) of Zr–TiO_2_ was obtained at −0.45 V versus RHE, which exceeded that of the undoped TiO_2_ and Ce–TiO_2_. The work calls for researchers to pay more attention to dopants with the same oxidation state. Very recently, Wang and co‐workers prepared Pt‐doped hollow iron phosphide nanorods (Pt–FeP/C) via derivatizing Pt‐containing MIL‐88A.^[^ ^177^ ^]^ DFT calculations revealed that Fe was the catalytic active center, P was an accelerator and proton provider for water splitting, and Pt doping effectively accelerated proton transfer. Compared with undoped materials, Fe doping will effectively reduce the energy barrier of the rate‐determining step (*N_2_ → *N_2_H). In the end, the Pt–FeP/C obtained an NH_3_ yield of 10.22 µg h^−1^ cm^−2^ and an excellent FE of 15.3% at relatively low voltage (−0.05 V), which was the highest NH_3_ yield among the iron‐based catalysts.

Reasonable selection of codopants can induce additional active sites for desirable catalytic performance, so heteroatom codoping is also an expected strategy. Yan and co‐workers prepared Fe,Mo‐codoped hollow porous carbon nanorods (Fe,Mo–N/C) using zinc oxide nanorods as templates (Figure 10g).^[^ ^176^ ^]^ Fe,Mo–N/C obtained the highest NH_3_ yield of 1.52 × 10^−6^ mol h^−1^ cm^−2^ and the best FE of 14.2%, both of which were better than those of single Fe‐ or Mo‐doped hollow porous carbon nanorods (Figure 10h). Such excellent performance should be attributed to the promotion of N≡N cleavage by Fe and Mo as active sites, the enhancement of N_2_ adsorption by high pyridine nitrogen content in the material, and the high specific surface area of the hollow structural materials to enhance the exposure of active sites. Very recently, Chen and co‐workers synthesized Ni–Fe‐doped MoS_2_ nanocages (Ni–Fe@MoS_2_ NCs) using a self‐template method for NRR.^[^ ^178^ ^]^ XPS confirmed that Ni–Fe codoping caused a large number of Mo—S bonds to break and then produced highly oxidized species, as well as abundant sulfur defects. DFT calculations showed that the active site of Ni_0.3_Fe_0.7_ was key to accelerating electron transfer in NRR reaction. The spin‐polarized density of state revealed that N_2_ adsorbed on the active site of Ni_0.3_Fe_0.7_ can easily obtain electrons provided by the S 2p and Mo 3d orbitals during the NRR process. As expected, Ni–Fe@MoS_2_ NCs obtained an excellent NH_3_ yield of 128.17 µg h^−1^ mg^−1^_cat._ and a good FE of 11.34% at −0.3 V versus RHE.

In summary, doping heteroatoms can effectively adjust the electronic structure to enhance the N_2_ adsorption, which inspires researchers to regulate efficient NRR catalysts through doping engineering. However, its development suffers from the following drawbacks. First of all, it is difficult to achieve a controllable doping amount and precise anchoring position. It is therefore urgently needed to develop reliable synthetic methods that can be mass‐produced and possess universal applicability. This is particularly important for N‐doped or N‐containing materials to prevent the effects of doping on their stability and thus to avoid the generation of pollution sources that affect NRR results. Second, it is challenging to explore the relationship between doping types and activities and to precisely synthesize specific doping types. For example, N‐doped carbon materials will produce nitrogen‐containing functional groups such as pyridine nitrogen, pyrrole nitrogen, and graphite nitrogen. However, some studies have found that only pyridine nitrogen and pyrrole nitrogen are the actual active sites for catalysis. Therefore, it is important to investigate the real active sites and formulate precise synthetic methods before doping. In addition, some codoping strategies have achieved remarkable results, which encourage researchers to invest more efforts in exploring the synergistic effects of heteroatoms in the future.

#### 4.3.2 Vacancy Engineering

Vacancy engineering is regarded as another important strategy to adjust the physical or chemical properties of materials. It has been proven that the introduction of vacancies can tune the electronic structure, increase the electron trapping capacity, and improve the surface adsorption capacity. In addition, vacancies with plenty of localized electrons are also good anchoring sites for single atoms (SAs). Vacancy can be simply divided into anion vacancies and cation vacancies according to their different oxidation states. Anion vacancies, such as oxygen vacancies,^[^ ^179^ ^]^ sulfur vacancies,^[^ ^180^ ^]^ and nitrogen vacancies,^[^ ^181^ ^]^ have been widely studied because of their low formation energy, while cation vacancies have not been paid much attention because of their higher formation energy barriers relative to anion vacancies. At present, in addition to the doping process described in the previous section that can create vacancies, other effective methods, such as heat treatment in an oxygen‐deficient environment,^[^ ^182^ ^]^ chemical reduction,^[^ ^183^ ^]^ and plasma treatment have also been developed.^[^ ^184^ ^]^

**Figure 11:**
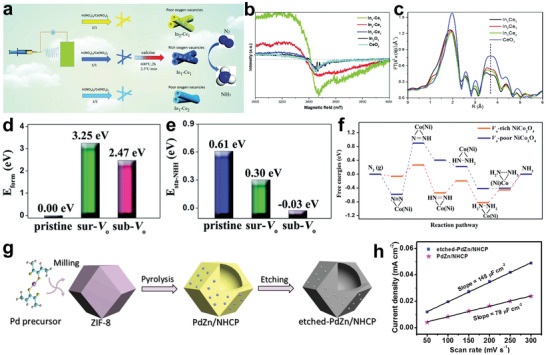
a) Schematic illustration of the formation of the V_o_‐rich In_2_O_3−_ *_x_*/CeO_2−_ *_y_*. b) EPR spectra of the different catalysts. c) EXAFS signals in *R*‐space of the catalysts. Reproduced with permission.^[^ ^185^ ^]^ Copyright 2020, Royal Society of Chemistry. d) Formation energy of oxygen vacancy for different catalyst models. e) Adsorption energy of NNH groups in different catalyst models. f) Free energy diagram of V_o_‐rich NiCo_2_O_4_@HNCP. Reproduced with permission.^[^ ^186^ ^]^ Copyright 2020, Royal Society of Chemistry. g) Schematic diagram of the formation process of etched‐PdZn/NHCP. h) Capacitive currents versus scan rates for different catalysts. Reproduced with permission.^[^ ^188^ ^]^ Copyright 2020, Elsevier.

Oxygen vacancy (V_o_) has been widely studied due to its low formation energy. Previous studies have shown that the V_o_s on the surface of metal oxides are the active centers for N_2_ adsorption and activation. Very recently, Ye and co‐workers found that In_2_O_3−_ *_x_*/CeO_2−_ *_y_* with V_o_s can stretch the N≡N adsorbed on its surface through DFT calculations, which would lower the energy barrier and benefit the NRR reaction.^[^ ^185^ ^]^ They prepared In_2_O_3−_ *_x_*/CeO_2−_ *_y_* nanotubes (In_2_–Ce_1_, In_1_–Ce_1_, In_1_–Ce_2_) as NRR catalysts with different In/Ce ratios (2:1, 1:1, 1:2) by electrospinning (**Figure** **11**a). In_1_–Ce_1_ had the highest specific surface area, the largest electrochemical active surface area (ECSA), and the smallest charge transfer resistance, which were beneficial to expose more active sites and increase the selectivity of NRR. As shown in Figure 11b,c, both EPR spectrum and Fourier transform extended X‐ray absorption fine structure (EXAFS) spectrum confirmed the existence of V_o_s of In_1_–Ce_1_. The well‐designed In_1_–Ce_1_ exhibited the highest NH_3_ yield of 26.1 µg h^−1^ mg^−1^_cat._ and the maximum FE of 16.1% at −0.3 V versus RHE.

In addition, Liu and co‐workers have designed several V_o_‐rich spinel nanosheets loaded on hollow nitrogen‐doped carbon polyhedra (HNCP) under the guidance of DFT calculations.^[^ ^186^ ^]^ Taking V_o_‐rich NiCo_2_O_4_ on HNCP (V_o_‐rich NiCo_2_O_4_@HNCP) as an example, both XPS and X‐ray absorption near edge structure spectra confirmed the existence of abundant V_o_s. In addition, they found that the annealing temperature would affect the content of V_o_s. The type of V_o_ was also an important factor in determining the catalytic activity. There were two types of oxygen, surface oxygen and subsurface oxygen, in the perfectly crystallized NiCo_2_O_4_ lattice. As shown in Figure 11d,e, subsurface oxygen vacancy (sub‐V_o_) had lower formation energy and higher adsorption capacity for NNH groups. In 0.1 m Na_2_SO_4_, the best NRR performance of V_o_‐rich NiCo_2_O_4_@HNCP (NH_3_ yield: 4.1 µg h^−1^ cm^−2^; FE: 5.3%) was obtained at −0.25 V versus RHE, which was better than V_o_‐rich NiCo_2_O_4_ bulk and V_o_‐poor NiCo_2_O_4_@HNCP (V_o_‐poor NiCo_2_O_4_@HNCP). Furthermore, theoretical calculations indicated that the NRR process on the catalyst surface may follow an associative alternating pathway. In comparison to V_o_‐poor NiCo_2_O_4_@HNCP, V_o_‐rich NiCo_2_O_4_@HNCP had lower free energy, suggesting that introducing vacancies was indeed beneficial to the NRR process (Figure 11f). The well‐designed V_o_s and hollow structures synergistically promoted the catalytic performance, as also confirmed in other spinel hollow structural materials (ZnCo_2_O_4_@HNCP and Co_3_O_4_@HNCP).

The presence of anion vacancies can effectively improve the NRR catalytic activity of catalysts, which motivates researchers to further explore cation vacancies. Wang and co‐workers prepared MoN nanocrystals with rich Mo vacancies anchored on N‐doped carbon (MV–MoN@NC) through utilizing SiO_2_ spheres as templates and etching treatment.^[^ ^187^ ^]^ In HRTEM image, a myriad of defects were found in MV–MoN@NC, which was subsequently confirmed by EPR and XPS spectra. In addition, MV–MoN@NC had the highest ECSA value and the best charge transfer ability, which indicated that Mo vacancy not only improved the catalytic activity but also enhanced the electrical conductivity. The hollow structure and vacancies synergistically worked on the cleverly designed MV–MoN@NC, showing much higher NH_3_ yield (76.9 µg h^−1^ mg^−1^_cat._) than MoN@NC (31.1 µg h^−1^ mg^−1^_cat._), MoN (9.2 µg h^−1^ mg^−1^_cat._), and NC (2.2 µg h^−1^ mg^−1^_cat._). The ^15^N isotope tracer experiment and DFT calculation confirmed that the reaction pathway of MV–MoN@NC was the MvK pathway.

Kuang and co‐workers prepared PdZn nanoparticles loaded on nitrogen‐doped hollow carbon polyhedrons (etched‐PdZn/NHCP) by pyrolyzing the MOF precursors and etching strategy (Figure 11g).^[^ ^188^ ^]^ Various characterization methods such as HRTEM, XPS, and EPR confirmed that the etched‐PdZn/NHCP contained a myriad of Zn vacancies, which would facilitate the capture and further activation of N_2_. The etching process not only removed ZnO nanoparticles in the material, but also increased the specific surface area of the material. Moreover, the ECSA of the material was also enhanced by etching, which was more conducive to the NRR reaction (Figure 11h). The synergistic effect of etching PdZn and NHCP with a large number of vacancies resulted in a remarkable FE of 16.9% and an NH_3_ yield of 5.28 µg h^−1^ mg^−1^_cat._ at −0.2 V versus RHE, far better than that of PdZn/NHCP (3.18 µg h^−1^ mg^−1^_cat._), commercial Pd/C (1.52 µg h^−1^ mg^−1^_cat._), and pure ZIF‐8‐derived N‐doped carbon (0.68 µg h^−1^ mg^−1^_cat._) under the same test conditions. The excellent performance of the designed catalyst was attributed to the following factors: the hollow structure that promoted electrolyte penetration and supported Pd/Zn nanoparticles, N‐doped carbon that improved the electron transfer rate, and defects that induced local electron‐rich activation of N_2_ after etching.

In conclusion, vacancy engineering is of great significance for improving the catalytic activity by improving the conductivity, regulating the electronic structures, and optimizing the active sites of catalysts. However, there are still some challenges that hinder the further development of vacancy engineering. 1) At present, most of the studies focus on the oxygen vacancies and nitrogen vacancies of anion vacancies, while other types of anion vacancies are not well explored. Similarly, from the effective improvement of electronic structure caused by anionic vacancies, it can be inferred that cationic vacancies are also promising alternative strategies that deserve extensive exploration. At the same time, combining multiple vacancies in the same catalyst is also a possible way to synergistically improve the catalytic performance due to differences in electronegativity and atomic radius. 2) The intrinsic relationship between vacancy content and catalytic activity is still unclear, so it is necessary to measure the vacancy contents correctly. At present, XPS is commonly used to identify the vacancy contents. However, XPS is a surface characterization method, and the fact that fitting of the vacancy contents is subjective makes it difficult to accurately reveal the actual contents of vacancy. Scanning tunneling microscope can intuitively observe the existence of vacancies, but it can only be used in a small area and cannot comprehensively count the vacancy contents. Therefore, it is necessary to develop advanced characterization techniques to determine the type and vacancy concentrations. 3) Last but not the least, in order to maintain the durability of the catalyst, the vacancies as active sites should stay stable for a long term, therefore, the stability of vacancies should be also considered when manufacturing defective catalysts.

#### 4.3.3 Single Atom Engineering

Recently, scientists have developed atomic‐scale dispersed single atomic catalysts (SACs).^[^ ^189^, ^190^ ^]^ Atom‐level exposure of SACs makes full use of individual atoms, not only improving quality and yield, but also effectively saving costs, especially for precious metals. At present, SACs have made considerable progress in the field of catalysis, such as oxygen reduction reaction,^[^ ^191^ ^]^ carbon dioxide reduction reaction,^[^ ^192^ ^]^ oxygen evolution reaction,^[^ ^193^ ^]^ HER,^[^ ^194^ ^]^ and NRR.^[^ ^195^ ^]^ However, their surface energy is high due to the reduced size, making it necessary to find a suitable substrate to anchor them. Carbon‐based materials with high conductivity and large specific surface area are widely used as carriers for SACs. Among them, carriers with hollow characteristics such as CNTs and MOF derivatives are favored.^[^ ^196^, ^197^ ^]^

**Figure 12:**
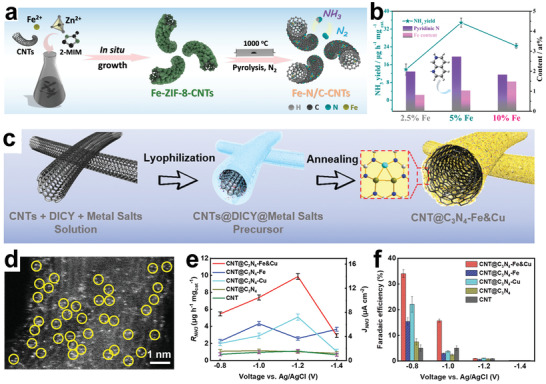
a) Scheme illustration of the synthesis of Fe–N/C–CNTs. b) Correlation between NH_3_ yields and different N contents of Fe–N/C–CNTs with different amounts of Fe. Reproduced with permission.^[^ ^197^ ^]^ Copyright 2019, American Chemical Society. c) Schematic illustration of preparation and morphology of CNT@C_3_N_4_–FeandCu. d) STEM image of CNT@C_3_N_4_–FeandCu. e) NH_3_ yields and partial current densities of different catalysts. f) FEs of different catalysts. Reproduced with permission.^[^ ^199^ ^]^ Copyright 2020, Wiley‐VCH.

Fe–N_3_ is considered to be a potent active site for NRR. As a proof‐of‐concept experiment, Cui and co‐workers synthesized Fe–N/C–CNTs as NRR catalysts by pyrolyzing the iron‐doped ZIF‐8 dispersed on CNTs (**Figure** **12**a).^[^ ^197^ ^]^ Fe SAs played a decisive role for enhancing NRR performance, which was confirmed by N_2_‐TPD. The results showed that Fe–N/C–CNTs not only obtained enhanced physical adsorption capacity, but also achieved superior nitrogen chemical adsorption capacity, which confirmed the excellent binding force of N_2_ and Fe SAs. In addition, ZIF‐8 crystals were uniformly loaded on the surface of CNTs, which resulted in a high specific surface area of Fe–N/C–CNTs and effectively increased the catalytic active area. At −0.2 V versus RHE, the ingeniously designed Fe–N/C–CNTs showed the highest NH_3_ yield of 34.83 µg h^−1^ mg^−1^_cat._ and the corresponding FE of 9.28%, which were higher than N‐doped carbon on CNTs (NC–CNTs) and CNTs. Fe–N/C–CNTs were deactivated by SCN^−^ toxicity under acidic conditions and reactivated by dissociation of SCN^−^ under alkaline conditions, which agreed with the hypothesis that Fe–N_3_ was the active site of NRR. Furthermore, the NH_3_ yield depended on not only the iron content, but also the pyridine nitrogen content, which indicated that the pyridine nitrogen was the anchor point of Fe–N_3_ (Figure 12b). SAs are known as ideal N_2_ adsorption sites, so more other kinds of SAs based on hollow structural materials should be manufactured. The development of SA anchor sites is another priority, for it is not only a prerequisite for SAs’ stability but also a determining factor of SAs’ total number.

Meanwhile, SAs can also synergize with nanoparticles to promote NRR. The theoretical study of Wang and co‐workers showed that Mo_2_C and MoSAs exhibited the selectivity of HER and NRR, respectively.^[^ ^198^ ^]^ Accordingly, Mo_2_C was expected to obtain a large amount of *H provided by MoSAs to activate N_2_, which would enable the catalyst complex combining Mo_2_C and MoSAs to improve selectivity and activity at the same time. Subsequently, MoSAs and Mo_2_C were assembled into N‐doped carbon nanotubes (MoSAs–Mo_2_C/NCNTs) as NRR cathode materials. MoSAs–Mo_2_C/NCNTs exhibited the highest NH_3_ yield of 16.1 µg h^−1^ cm_cat._^−2^ and the maximum FE of 7.1%, which were better than the single component MoSAs/NCNTs (3.3 µg h^−1^ cm_cat._^−2^, 1.4%) and Mo_2_C/NCNTs (4.3 µg h^−1^ cm_cat._^−2^, 3.6%). Although the synergy is proposed by combining the experimental results with the modeling calculations, the conclusion derivation process is often not comprehensive enough, which calls for the utilization and advancement of in situ characterization methods (such as in situ X‐ray absorption spectroscopy, in situ microscopy, etc.).

In addition to single metal SAs, as shown in Figure 12c, Liang and co‐workers used C_3_N_4_ supported on CNT to anchor Fe and Cu SAs (CNT@C_3_N_4_–FeandCu) as an efficient NRR catalyst.^[^ ^199^ ^]^ Scanning transmission electron microscopy (STEM) confirmed that the surface of the material contained a large number of SAs with most of them being in a triplet state (Figure 12d). The distance between SAs in the triplet state was 1.8–2.5 Å, very close to the bond length of N≡N. Such distance was enough to produce a synergistic effect between SAs as supported by the theoretical calculations.^[^ ^200^ ^]^ When a N_2_ is adsorbed on an active site with three metal atoms, the three metal atoms can stabilize N_2_ at the same time, which can avoid the formation of strong N–metal bonds, thereby realizing low‐energy NH_3_ desorption. As shown in Figure 12e,f, the as‐prepared CNT@C_3_N_4_–FeandCu obtained the best NH_3_ yield of 10.27 µg h^−1^ mg^−1^_cat._ and an excellent FE of up to 34%, which were better than Fe or Cu SAs analogues (CNT@C_3_N_4_–Fe and CNT@C_3_N_4_–Cu). The DFT calculations showed that the PDS of CNT@C_3_N_4_–FeandCu (*N_2_ → *N_2_H, Δ*G* = 0.58 eV) was better than that of CNT@C_3_N_4_–Fe (*NH → *NH_2_, Δ*G* = 0.71 eV) and CNT@C_3_N_4_–Cu (N_2_ → *N_2_, Δ*G* = 0.86 eV). This bimetallic SAs with triplet state enhanced the adsorption of N_2_ and achieved a reaction process with a lower energy barrier, which is of great significance for low‐cost NH_3_ production under mild conditions.

In short, SAs are considered to be good NRR catalyst candidates due to their unique electronic structure, uniform low‐coordination environment, and atomically dispersed active sites, which can maximize the exposure of active sites. Anchoring SAs on the surface of hollow structural materials through strong coordination can effectively prevent their agglomeration, so as to efficiently and stably catalyze the conversion of N_2_ to NH_3_. Such excellent catalytic performance has led researchers to develop SACs with higher loading amounts. However, it is important to note that improving the anchor point of SAs is a prerequisite and the synergistic effect of SAs and other active components is worth further investigation.

#### 4.3.4 Other Engineering

**Figure 13:**
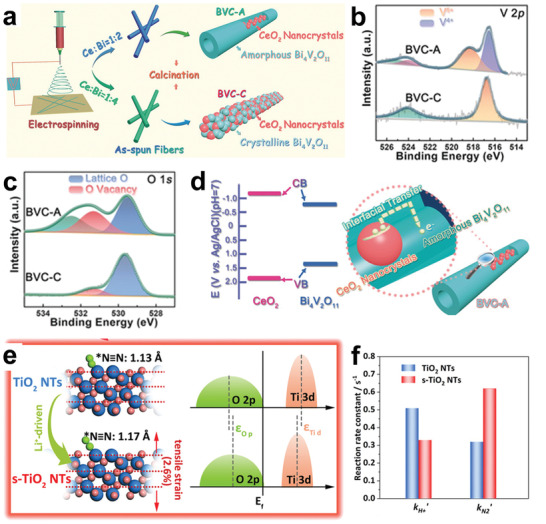
a) Schematic illustration of the preparation of BVC‐A and BVC‐C electrocatalysts. b) XPS spectra of V 2p. c) XPS spectra of O 1s. d) Band alignment of CeO_2_ and Bi_4_O_11_ and scheme of interfacial charge transfer of BVC‐A. Reproduced with permission.^[^ ^203^ ^]^ Copyright 2018, Wiley‐VCH. e) Bond length of adsorbed N_2_ on different catalysts and the relative density of states. f) Reaction rate constants of H^+^ and N_2_ adsorption on Ti^3+^ site in different catalysts. Reproduced with permission.^[^ ^28^ ^]^ Copyright 2020, Wiley‐VCH.

Amorphous materials have a disordered arrangement of atoms and highly unsaturated coordination sites, which will produce dangling bonds, introduce a large number of defects, and effectively enhance catalytic activity.^[^ ^201^, ^202^ ^]^ As shown in **Figure** **13**a, Yu and co‐workers prepared a Bi_4_V_2_O_11_/CeO_2_ composite (BVC‐A) with an amorphous phase by electrospinning.^[^ ^203^ ^]^ They found that CeO_2_ could hinder heat transfer, and by adjusting the ratio of Ce to Bi, it might be possible to produce materials with different crystal shapes. When Ce:Bi was 1:2, a hollow amorphous Bi_4_V_2_O_11_ nanofiber and CeO_2_ nanocrystal hybrid (BVC‐A) were produced, while when Ce:Bi was 1:4, a Bi_4_V_2_O_11_ nanocrystal and CeO_2_ nanocrystal hybrid (BVC‐C) were produced. As shown in Figure 13b,c, XPS spectroscopy demonstrated that amorphization could also induce oxygen vacancies in addition to introducing tetravalent defects V. In addition, CeO_2_ would also form a heterojunction with amorphous Bi_4_V_2_O_11_, as shown in Figure 13d. The resulting Bi_4_V_2_O_11_/CeO_2_ composite displayed a type I band alignment, which would facilitate the electron transfer from CeO_2_ to Bi_4_V_2_O_11_. BVC‐A displayed the optimum NH_3_ production rate of 23.21 µg h^−1^ mg^−1^_cat._ and the highest FE of 10.16% at −0.2 V versus RHE. BVC‐A obtained more excellent NRR activity than BVC‐C and pure CeO_2_ and Bi_4_V_2_O_11_, thanks to a multitude of defect sites from the amorphousness, the large active surface area exposed by the hollow structure, and the acceleration of electron transport by heterostructure.

Lattice strain is a strategy to change the surface electronic structure and affect the catalytic activity by adjusting the distance between surface atoms.^[^ ^204^, ^205^ ^]^ Yu and co‐workers investigated the influence of morphology on catalytic performance and found that the tubular materials can effectively increase the yield because they have both the high selectivity and high surface area of annular surfaces.^[^ ^28^ ^]^ Furthermore, the theoretical calculations found that the Li^+^ intercalation and deintercalation were able to stretch the N≡N of N_2_ adsorbed on Ti^3+^ sites and reduce the formation energy of oxygen vacancies for further stabilizing Ti^3+^, as shown in Figure 13e. Through theoretical inferences, they prepared lattice‐strained TiO_2_ nanotubes (s‐TiO_2_ NTs) by utilizing electrochemical Li^+^ intercalation and deintercalation strategies. The reaction rate constants of N_2_ and H^+^ adsorbed on s‐TiO_2_ NTs and TiO_2_ NTs were measured by in situ surface interrogation scanning electrochemical microscopy technique, indicating the higher NRR selectivity of N_2_ and H^+^ adsorbed on s‐TiO_2_ NTs (Figure 13f). The NH_3_ yield (16.67 µg h^−1^ mg^−1^_cat._) and FE (26%) of the elaborately prepared s‐TiO_2_ NTs showed a substantial increase compared to TiO_2_ NTs and TiO_2_ thin films. DFT calculations revealed that s‐TiO_2_ NTs were more conducive to N_2_ adsorption, so they were first protonated at lower energy as a rate‐determining step, and then NRR is effectively performed along the associative distal pathway. This interesting work encourages researchers to investigate the effects of metal ion intercalation and deintercalation strategies on lattice strain. In short, amorphous and lattice strains are effective strategies for optimizing hollow structural catalysts and deserve in‐depth study.

## 5 Summary and Outlook

The electrocatalytic conversion of N_2_ to NH_3_ under environmental conditions has become an attractive subject in line with the background of carbon neutral. As promising catalysts, the hollow structural materials have fascinating merits, including large specific surface area, high atomic utilization, reliable structural stability, low mass and charge transfer pathway, and the confined cavity on reactants, which make them to be excellent candidates for NRR electrocatalysts. In this review, the latest research progress of NRR hollow structural electrocatalysts under mild circumstances from intrinsic, mesoscopic to microscopic regulations is reviewed (**Table** **1**). Here, the main challenges faced in pursuit of obtaining extraordinary and efficient hollow structural electrocatalysts that can meet the requirements of industrial grades are summarized.

**Table 1:** A summary of reported hollow structural materials as NRR electrocatalysts in recent years and their catalytic performance

	Catalyst	Electrolyte	NH_3_ yield rate	Faradaic efficiency [%]	Potential ([V] vs RHE)	Ref.
Intrinsic	VO_2_ microspheres	0.1 m Na_2_SO_4_	14.85 µg h^−1^ mg^−1^_cat._	3.97	−0.7	[128]
	Bi_2_MoO_6_ spheres	0.1 m HCl	20.46 µg h^−1^ mg^−1^_cat._	8.17	−0.6	[129]
	Bi nanospheres	0.1 m Na_2_SO_4_	23.4 ± 1.3 µg h^−1^ mg^−1^_cat._	19.8 ± 1.1	−0.4	[130]
	Cr_2_O_3_ microspheres	0.1 m Na_2_SO_4_	25.3 µg h^−1^ mg^−1^_cat._	6.78	−0.9	[131]
	MoPi microspheres	0.1 m KOH	18.66 µg h^−1^ mg^−1^_cat._	9.04	−0.2	[135]
	CoPi microspheres	0.1 m KOH	16.48 µg h^−1^ mg^−1^_cat._	4.46	−0.2	[136]
	CoPc nanotubes	0.1 m HCl	107.9 µg h^−1^ mg^−1^_cat._	27.7	−0.3	[137]
	Au nanocages	0.5 m LiClO_4_	3.9 µg cm^−2^ h^−1^	30.2	−0.5, −0.4	[31]
	Au nanocage‐715	0.5 m LiClO_4_	3.74 µg cm^−2^ h^−1^	35.9	−0.4	[138]
	CrN nanocube	0.1 m HCl	31.11 µg h^−1^ mg^−1^_cat._	16.6	−0.5	[206]
Mesoscopic	FePc/O‐MWCNT	0.1 m HCl	36 µg h^−1^ mg^−1^_cat._	9.73	−0.3	[144]
	NCNT/Fe_3_C	0.1 m KOH	15.508 µg h^−1^ mg^−1^_cat._	2.72	−0.4	[145]
	CuCo_2_S_4_/MWCNT	0.1 m Na_2_SO_4_	137.5 µg h^−1^ mg^−1^_cat._	8.7	−0.5	[146]
	Ru–PEI@MWCNTs	0.1 m KOH	188.9 µg h^−1^ mg^−1^_cat._	30.93	−0.1	[147]
	Mn_3_O_4_/b‐TiO_2_	0.1 m KOH	1.61 × 10^−10^ mol s^−1^ cm^−2^	25.2	−0.45, −0.35	[148]
	BP@SnO_2−_ *_x_*	0.1 m Na_2_SO_4_	48.87 µg h^−1^ mg^−1^_cat._	14.6	−0.4	[149]
	NiCoS/C	0.1 m Li_2_SO_4_	58.5 µg h^−1^ mg^−1^_cat._	12.9	−0.2, 0	[161]
	CoVP@NiFeV–LDHs	0.05 m H_2_SO_4_	27.2 µg h^−1^ mg^−1^_cat._	13.8	−0.3	[162]
	Ni@NCNTs	0.1 m HCl	53.88 µg h^−1^ mg^−1^_cat._	7.3	−0.5, −0.3	[163]
	PdO/Pd/CNTs	0.1 m NaOH	18.2 µg h^−1^ mg^−1^_cat._	11.5	0, 0.1	[164]
	C@NiO@Ni	0.1 m KOH	43.15 µg h^−1^ mg^−1^_cat._	10.9	−0.7	[165]
	Au_3_Cu@Cu	0.1 m Na_2_SO_4_	33.97 µg h^−1^ mg^−1^_cat._	21.41	−0.2	[167]
	Co_3_O_4_@NCs	0.05 m H_2_SO_4_	42.58 µg h^−1^ mg^−1^_cat._	8.5	−0.2	[166]
	Au–Ag–Pd‐850	0.5 m LiClO_4_	13.74 µg h^−1^ mg^−1^_cat._	48.94	−0.3	[207]
	Co–MoS_2_/N@C	0.1 m Na_2_SO_4_	129.93 µg h^−1^ mg^−1^_cat._	11.21	−0.4	[208]
	1T′‐MoS_2_/TiO_2_	0.1 m Na_2_SO_4_	29.62 µg h^−1^ mg^−1^_cat._	24.9	−0.75, −0.65	[209]
Microscopic	S–CNS	0.1 m Na_2_SO_4_	19.07 µg h^−1^ mg^−1^_cat._	7.47	−0.7	[171]
	O–C microtubes	0.1 m HCl	25.12 µg h^−1^ mg^−1^_cat._	9.1	−0.85, −0.80	[210]
	O–CNT	0.1 m LiClO_4_	32.33 µg h^−1^ mg^−1^_cat._	12.50	−0.4	[211]
	P–CNTs	0.25 m LiClO_4_	24.4 µg h^−1^ mg^−1^_cat._	12.5	−1.1, −0.3	[172]
	Fe–TiO_2_	0.1 m Na_2_SO_4_	43.14 µg h^−1^ mg^−1^_cat._	16.35	−0.7	[174]
	Zr–TiO_2_	0.1 m KOH	8.90 µg h^−1^ mg^−1^_cat._	17.3	−0.45	[175]
	Pt–FeP/C	0.1 m KOH	54.75 µg h^−1^ mg^−1^_cat._	15.3	−0.1, −0.05	[177]
	Fe,Mo–N/C	0.1 m Na_2_SO_4_	38.76 µg h^−1^ mg^−1^_cat._	14.2	−0.1	[176]
	NiFe–MoS_2_	0.1 m Na_2_SO_4_	106.59 µg h^−1^ mg^−1^_cat._	7.15	−0.3, −0.2	[178]
	In_2_O_3−_ *_x_*/CeO_2−_ *_y_*	0.1 m KOH	26.1 µg h^−1^ mg^−1^_cat._	16.1	−0.3	[185]
	NiCo_2_O_4_@HNCP	0.1 m Na_2_SO_4_	17.8 µg h^−1^ mg^−1^_cat._	5.3	−0.25	[186]
	MV–MoN@NC	0.1 m HCl	76.9 µg h^−1^ mg^−1^_cat._	6.9	−0.2	[187]
	etched‐PdZn/NHCP	0.1 m phosphate‐buffered saline	5.28 µg h^−1^ mg^−1^_cat._	16.9	−0.2	[188]
	Fe–N/C–CNTs	0.1 m KOH	34.83 µg h^−1^ mg^−1^_cat._	9.28	−0.2	[197]
	MoSAs–Mo_2_C/NCNTs	0.005 m H_2_SO_4_ + 0.1 m K_2_SO_4_	16.1 µg h^−1^ mg^−1^_cat._	7.1	−0.25, −0.2	[198]
	CNT@C_3_N_4_–FeandCu	0.25 m LiClO_4_	10.27 µg h^−1^ mg^−1^_cat._	34	−1.2, −0.8	[199]
	Bi_4_O_11_/CeO_2_	0.1 m HCl	23.21 µg h^−1^ mg^−1^_cat._	10.16	−0.2	[203]
	s‐TiO_2_	0.1 m HCl	16.67 µg h^−1^ mg^−1^_cat._	26	−0.6, −0.45	[28]
	CoP	1.0 m KOH	10.78 µg h^−1^ mg^−1^_cat._	7.36	−0.4, 0	[212]
	C@CoFe_2_O_4−_ *_x_*	0.1 m Na_2_SO_4_	30.97 µg h^−1^ mg^−1^_cat._	11.65	−0.4	[213]

**i) Hollow structural catalyst synthesis**: Hollow materials with different structural features are usually synthesized by hard template, soft template, and self‐template/template‐free methods, each of which has its own advantages and disadvantages. However, there is still a long way to go to obtain the hollow materials with precise and controllable structures. Here, the following suggestions are proposed in order to develop a “green” synthetic method. 1) Factors such as cost saving, environment‐friendly, and large‐scale production should be considered in the process of developing new strategies to achieve industrial applications instead of being limited to laboratory research; meanwhile, the synthetic methods of using highly corrosive and toxic chemicals should be optimized. 2) It is meaningful to control the exposure number of active spots in hollow structural materials because that the number of active sites is generally proportional to the catalytic activity. Therefore, the size, morphology, pore structure, and shell thickness of the hollow structural material should be properly regulated to capture reactants and activate nitrogen molecules. 3) The synthesis of hollow structure models that can define active components is conducive to clarifying the reaction mechanism, and has profound guiding significance for future design and synthesis.

**ii) Intrinsic regulation of hollow structural catalysts**: High specific surface area and abundant cavities of the intrinsic hollow structural material provide highly unsaturated coordination and highly accessible surface sites for the catalytic reaction, guaranteeing high apparent activity. It is worth noting that the inner surface of the hollow structural material is less affected by the capping agent, so it may exhibit higher catalytic activity than the outer surface. Abundant cavities can limit diffusion of reaction intermediates and increase their collision possibility, thus increasing the probability of being captured by active sites. In addition, the stability of the hollow structures can prevent the aggregation and deactivation of active sites, making the catalysts more durable. These unique advantages promise the hollow structural materials to be strong candidates for NRR catalysts. Nevertheless, in order to obtain better catalytic activity, the shell thickness and pore size of hollow structural materials should be carefully designed. The thinner the shell thickness, the greater the atomic utilization and the larger the specific surface area, with the structural stability being well retained. The extreme small pore size would hinder the diffusion of the reactants, while large pore size would damnify specific surface area and also make it difficult to restrict the reactants.

**iii) Mesoscopic regulation of hollow structural catalysts**: From the mesoscopic point of view, the hollow structural materials could be used as good carriers due to their highly unsaturated coordination sites and reliable structural stability, which can increase N_2_ adsorption sites by anchoring other components. First of all, the hollow structural materials can uniformly scatter nanoparticles or other molecules, and in this way, the supported components can be prevented from agglomerating during catalysis, thereby improving the durability of the catalysts. Second, the hollow structural materials are capable of constructing heterostructures with other components and forming heterogeneous interfaces, which are conducive to enhancing the conductivity, promoting the adsorption of reactants, and accelerating the interfacial electron transfer. Third, some unstable active ingredients can improve their stability by forming core–shell heterostructures. Although in most cases mesoscopically regulated hollow structural materials are used as carriers, the activity of the hollow structural materials themselves should not be ignored. The performance of catalysts is not simply superimposed by hollow structural materials and other components, so we should not just summarize the active ingredients from synergistic effects without giving detailed evidence. Therefore, it is necessary to design control experiments ingeniously based on the advanced theoretical calculations to further distinguish their respective contributions.

**iv) Microscopic regulation of hollow structural catalysts**: From the microscopic point of view, the local coordination environment of hollow structural materials can be adjusted through the modification of atomic structures to improve catalytic activity. The strategy of introducing heteroatoms through doping could redistribute the electron density of the structures, thereby forming surface defects to improve the binding ability with N_2_. The fabrication of vacancies could increase the conductivity, tune the electronic structure, and optimize the active sites. The hollow structural material used to anchor SAs can not only increase the active sites, but also form electronic interactions generated by metal–substrate coordination that often produce impressive catalytic performance. In addition, amorphous and lattice strains, which have not been widely concerned, are also effective strategies for adjusting hollow structural materials. Although many microscopic regulation strategies have been developed that can effectively improve the catalytic performance of hollow structural catalysts, there are still problems in various strategies, such as the precise regulation of defect concentrations, and the precise synthesis in specific coordination environments. Meanwhile, the internal mechanism of various strategies to promote the efficient implementation of NRR is still unclear. In conclusion, there is an urgent need to develop microscopic regulation strategies with universality and controllability, and to further explore their respective roles in the catalytic process by combining advanced characterization techniques with theoretical calculations.

In short, hollow structural materials are excellent electrocatalysts for the conversion of N_2_ to NH_3_ under environmental conditions. In this review, the latest developments in the hollow structural catalysts have been reviewed, focusing on synthetic methods and regulating strategies. In order to design the catalyst more rationally, suggestions for optimizing synthetic methods and a top‐down fine regulating strategy have been proposed. It is anticipated to obtain more active, selective and stable hollow structural catalysts for the efficient production of NH_3_ in the future and even for industrial applications.

## Conflict of Interest

The authors declare no conflict of interest.
